# Bayesian optimisation for efficient parameter inference in a cardiac mechanics model of the left ventricle

**DOI:** 10.1002/cnm.3593

**Published:** 2022-04-07

**Authors:** Agnieszka Borowska, Hao Gao, Alan Lazarus, Dirk Husmeier

**Affiliations:** ^1^ School of Mathematics and Statistics University of Glasgow Glasgow UK

**Keywords:** biomechanical model calibration, global optimisation, Holzapfel‐Ogden constitutive law, statistical inference

## Abstract

We consider parameter inference in cardio‐mechanic models of the left ventricle, in particular the one based on the Holtzapfel‐Ogden (HO) constitutive law, using clinical in vivo data. The equations underlying these models do not admit closed form solutions and hence need to be solved numerically. These numerical procedures are computationally expensive making computational run times associated with numerical optimisation or sampling excessive for the uptake of the models in the clinical practice. To address this issue, we adopt the framework of Bayesian optimisation (BO), which is an efficient statistical technique of global optimisation. BO seeks the optimum of an unknown black‐box function by sequentially training a statistical surrogate‐model and using it to select the next query point by leveraging the associated exploration‐exploitation trade‐off. To guarantee that the estimates based on the in vivo data are realistic also for high‐pressures, unobservable in vivo, we include a penalty term based on a previously published empirical law developed using ex vivo data. Two case studies based on real data demonstrate that the proposed BO procedure outperforms the state‐of‐the‐art inference algorithm for the HO constitutive law.

## Graphical abstract

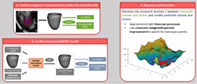

## 1 INTRODUCTION

The last decade has seen impressive progress in the mathematical modelling of soft‐tissue mechanics.^1^, ^2^ In particular, the specific application to cardiac mechanics^3^ promises to improve our understanding of cardiac physiology and pathology. A recent case–control study^4^ involving patients diagnosed with myocardial infarction (MI) and healthy volunteers has demonstrated that the biophysical parameters of a cardio‐mechanic model of the left ventricle (LV) of the heart can be non‐invasively estimated from cardiac magnetic resonance (CMR) scans, and that the parameters thus inferred allow for an accurate classification of subject disease status. However, the cardio‐mechanic equations do not admit a closed‐form solution and require computationally expensive numerical procedures based on finite element method (FEM).^5^ These procedures have to be repeated iteratively as part of a numerical optimisation or sampling procedure, leading to potentially excessive computational run times that discourage uptake of the methodology in the clinical practice.

We aim to address this difficulty by adopting the framework of Bayesian optimisation (BO), which has been particularly designed for optimisation problems where each evaluation of the objective function is expensive and the number of such evaluations has to be kept as low as possible. The main idea is to sequentially approximate the unknown objective function by a statistical model and use this model to identify the next query point, typically according to some exploration‐versus‐exploitation trade‐off criterion. In the literature the said statistical model is typically referred to as a *surrogate function* ^6^, ^7^ and Gaussian processes^8^ are commonly used in this context. (Surrogates are also called approximations, metamodels, response surface models or emulators.^9^ We reserve the latter term for pre‐trained approximations based on considerably larger numbers of points compared to those used in BO.) The trade‐off criterion is quantified by the so‐called *acquisition function*. Several strategies on how to specify the details of BO, in particular the acquisition function, have been proposed in the literature.^6^ In this study, we use a popular criterion called Expected Improvement^10^ as it admits a closed‐form expression for Gaussian process surrogates, is conceptually simple and performs well for a range of problems.^11^

To date, there have only been a few applications of BO to physiology and clinical decision support, noticeably published only recently. BO has been applied to improve personalised time series models for patient monitoring^12^ or for efficient human‐in‐the‐loop optimisation of wearable devices, for example powered prosthetics for walking assistance^13^ or soft exosuits.^14^ BO has also allowed for rapidly mapping residual network function in stroke^15^ or an efficient design of transcatheter aortic valve leaflets and optimisation of their geometry.^16^ BO has been also used in the context of computer‐aided (or robot‐assisted) surgery.^17^, ^18^ Another strand of literature has applied BO for efficient parameter inference for finite‐element problems, for example in haemodynamics,^19^ for learning material property of atherosclerotic carotid arteries,^20^ or in cardiology.^21^ The latter study of Fan et al.^21^ is most closely related to our work as it has combined finite element modelling and BO to characterise exercise‐induced myocardium growth to infer two unknown growth parameters. The main difference to our work is that Fan et al.^21^ focused on the estimation of two growth parameters affecting myocardial growth, whereas our work focuses on constitutive parameters determining the passive filling process in diastole.

Myocardial stiffness has been considered an important factor in heart failure patients with preserved ejection fraction,^22^, ^23^ which is thought to be caused by myocyte stiffening and interstitial fibrosis.^24^ To this end, a robust and fast estimation of myocardial passive stiffness is a key step for improved patient characterisation and treatment planning, which remains a great challenge in the cardiac modelling community despite continuous efforts in decades.^25^, ^26^, ^27^, ^28^, ^29^, ^30^, ^31^ The purpose of the present paper is to adapt the BO framework to the problem of inferring passive myocardial properties of healthy human LVs from in vivo measurements. In this study, the passive myocardial properties are characterised by an invariant‐based hyperelastic material model proposed by Holzapfel and Ogden^32^ (HO model), which has been widely used in cardiac modelling communities.^4^, ^33^, ^34^ The first attempt to address parameter estimation, also known as the inverse problem, in this context was made by Gao et al.^28^ These authors proposed an algorithmic, multi‐step procedure to infer the parameters of the HO model, in which in each step a subset of parameters was optimised with possibly different objective functions. The choice of particular subsets was based on expert knowledge of the myocardial properties of the LV. Our proposed BO approach is conceptually different, as BO iterations are run with respect to an a priori chosen parametrisation and objective function, thus requiring little expert knowledge.

To the best of our knowledge we are the first to introduce BO into cardiac mechanics for inferring unknown constitutive parameters. Our work contributes both to the global optimisation literature and to the application specific literature. Our first main contribution is developing a BO framework for the challenging problem of parameter inference in the HO model. Specifically, we propose modifications of the two components of BO, the target function and the acquisition function. The former modification consists in augmenting the target function by incorporating prior knowledge of the end‐diastolic volume‐pressure relationship from an empirical law established by Klotz et al.^35^ based on ex vivo data. This allows us to obtain more realistic myocardial properties not only at physiologically normal pressures but also at high pressures, usually not observed in vivo. Generally, the myocardium response to increasing pressure is nonlinear: it appears linear for typical low pressures observed in vivo for healthy subjects, and exponential for increasing high pressures, such as those observed for example for patients with hypertension. It can be challenging to infer those parameters that characterise the nonlinear stiffening effects based solely on in vivo data.

Our second main contribution is adapting the standard Expected Improvement criterion^10^ to the challenges of inference in the HO model. First, following previous studies,^36^, ^37^ we allow for unknown constraints in the parameter space by training a classifier based on a probity model with Gaussian process priors. Unknown constraints are related to those parameter regions that violate the physiological assumptions of cardio‐mechanic models, which is then demonstrated as crashes of the associated forward simulators. Second, next to the standard approach of developing a surrogate for the objective function as a whole, we develop an alternative paradigm, based on a *partial error surrogate*, in which we develop surrogates for individual squared error terms of the target function. This is motivated by a previous study^38^ in the context of parameter inference in the HO model, which reported a substantial improvement in accuracy in the emulation framework resulting from emulating a vector of outputs of the cardio‐mechanic model. Our proposed approach based on using a surrogate for the squared error terms instead of outputs has an advantage of being directly applicable to the Expected Improvement acquisition function, without requiring any approximation.

Our third main contribution is related to two empirical studies. First, we demonstrate that our proposed BO framework outperforms the state‐of‐the‐art algorithm of Gao et al.^28^ for parameter estimation in cardio‐mechanic models. Second, we show how incorporating the prior knowledge about volume‐pressure relationship (based on an empirical low established by Klotz et al.^35^ using ex vivo data) allows for physiologically more realistic parameter estimation consistent with the LV's characteristics in high pressure regimes, relevant for example for patients with hypertension or patients with diastolic heart failure.^22^

We note that in this paper we are concerned with Bayesian optimisation, which should not be confused with Bayesian inference. Bayesian inference is a statistical approach that quantifies uncertainty about model parameters via posterior distributions. Bayesian optimisation, on the other hand, is a computationally efficient global optimisation method. The word “Bayesian” in “Bayesian optimization” is related to the fact that methods from non‐parametric Bayesian statistics are used to approximate the objective function and quantify the uncertainty of the approximation. As opposed to Bayesian inference, uncertainty quantification in Bayesian optimisation is not an end in itself, but a means to another end: optimally trading off exploration and exploitation in the quest for a computationally efficient optimisation path. In fact, conceptually Bayesian optimisation is closer to classical, or frequentist, statistics, than to Bayesian statistics, as the frequentist approach is based on obtaining point estimates via optimising an underlying loss function.

The structure of this paper is as follows. Section 2 provides necessary details of the cardio‐mechanic model of interest and introduces the state‐of‐the‐art inference method.^28^ We discuss BO methodology, including the proposed extensions, in Section 3. Our three empirical studies are presented in Section 4. Section 5 concludes the paper and presents the outline for further research.

## 2 CARDIO‐MECHANIC MODEL OF THE LEFT VENTRICLE IN DIASTOLE

Biomechanical modelling of the LV in diastole is concerned with the process of diastolic filling, starting at early diastole and finishing at the end of diastole, which can be considered as a purely passive response. The nonlinear myocardial passive response means the myocardium is soft under lower pressure but becomes stiffer and stiffer under higher pressures because of gradually engaged collagen fibres.^32^ A few models of the LV biomechanics have been proposed in the literature, see a recent review by Chabiniok et al.^2^ In this work, we follow previous studies^28^, ^33^ and focus on the constitutive law proposed by Holzapfel and Ogden^32^ to describe passive myocardial response, in other words the myocardial stiffness. The adopted HO model is an invariant‐based incompressible hyperelastic constitutive law which takes into account the layered myofibre structure of the myocardium. Studies have demonstrated that it can realistically capture the key properties of the myocardium and at the same time is relatively easy to implement numerically.^39^ For these reasons it has been widely used in cardiac modelling communities^3^, ^4^, ^40^ The incompressible HO model is based on the following strain energy function
(1)

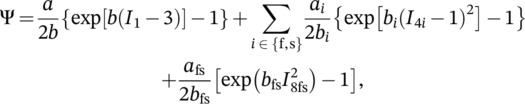

where $I_{i},i\in \left\{1,4f,4s,8\mathrm{fs}\right\}$ are invariants describing the deformation (corresponding to the matrix and fibre structure of the myocardium), and $a,b,a_{f},\;b_{f},\;a_{s},\;b_{s},\;a_{\mathrm{fs}},\;b_{\mathrm{fs}}$ are the eight constitutive parameters, which we collect in vector ϑ. The invariants in (1) are given as

(2) $$\begin{array}{ll} \;I_{1}=\mathrm{tr}\left(\mathbb{C}\right), & \;I_{4f}=f_{0}\cdot \left(\mathbb{C}f_{0}\right),\\ I_{4s}=s_{0}\cdot \left(\mathbb{C}s_{0}\right), & I_{8\mathrm{fs}}=f_{0}\cdot \left(\mathbb{C}s_{0}\right), \end{array}$$

where $\mathbb{C}=F^{T}F$ is the right Cauchy–Green deformation tensor, with F being the deformation gradient, while $f_{0}$ and $s_{0}$ are the myocyte and sheet directions, respectively, determined via a rule based approach, details can be found in Wang et al.^33^ Note that $f_{0}$ are $s_{0}$ are initial values hence are known before running the forward simulator. Given that the myocardium is incompressible, the Cauchy stress tensor can be determined from (1) as

(3) $$\sigma =\sum_{i=1,4f,4s,8\mathrm{fs}}\frac{\partial W}{\partial I_{i}}\frac{\partial I_{i}}{\partial F}F^{T}-pI,$$

in which p is a Lagrange multiplier to enforce the incompressibility constraint, and I is the identity matrix. The eight parameters in ϑ have the following interpretations: a and b describe the matrix response, $a_{f}$ and $b_{f}$ describe the contribution from the fibres along myocytes; $a_{s}$ and $b_{s}$ represent the contribution of the fibres along the sheet direction; $a_{\mathrm{fs}}$ and $b_{\mathrm{fs}}$ account for the shear effects in the fibre–sheet plane. Interested readers are referred to Holzapfel and Ogden^32^ for a detailed explanation of the HO model.

**FIGURE 1:**
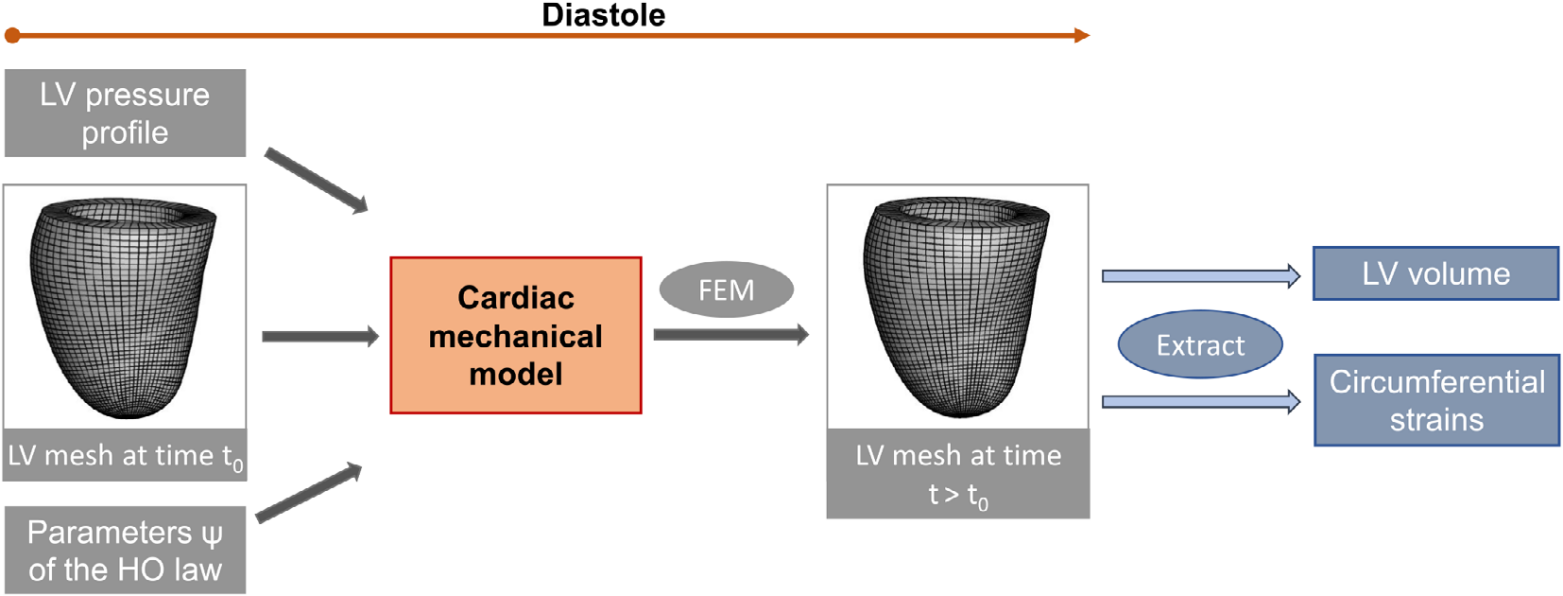
Inference pipeline in cardiac mechanic models

The quasi‐static cardio‐mechanics model in diastole is given by^41^

(4) $$\left.\begin{array}{ll} \nabla \cdot \sigma +b=0, & \text{in}\;\Omega ,\\ \;\sigma \cdot n=-p_{\text{endo}}n, & \text{in}\;\Gamma^{N},\\ \;u=\bar{u}, & \text{in}\;\Gamma^{D}, \end{array}\right\}$$

where **b** is the body force density per unit volume, $p_{\text{endo}}$ represents the LV cavity pressure, $\bar{u}$ is the vector of displacements at boundaries, Ω is the computational domain defined by the LV mesh with the boundary denoted as ∂Ω, **n** is the normal direction of ∂Ω, $\Gamma^{N}$and $\Gamma^{D}$ are the Neumann and Dirichlet boundaries, respectively, with $\Gamma^{N}\cup \Gamma^{D}=\partial \Omega$ and $\Gamma^{N}\cap \Gamma^{D}=\emptyset$. Solving (4) gives us the LV dynamics in response to the applied LV blood pressure, from which relevant quantities of interest can be readily extracted, such as the end‐diastolic LV volume and circumferential strains. Figure 1 illustrates the essential components of the cardio‐mechanic model in diastole.

**FIGURE 2:**
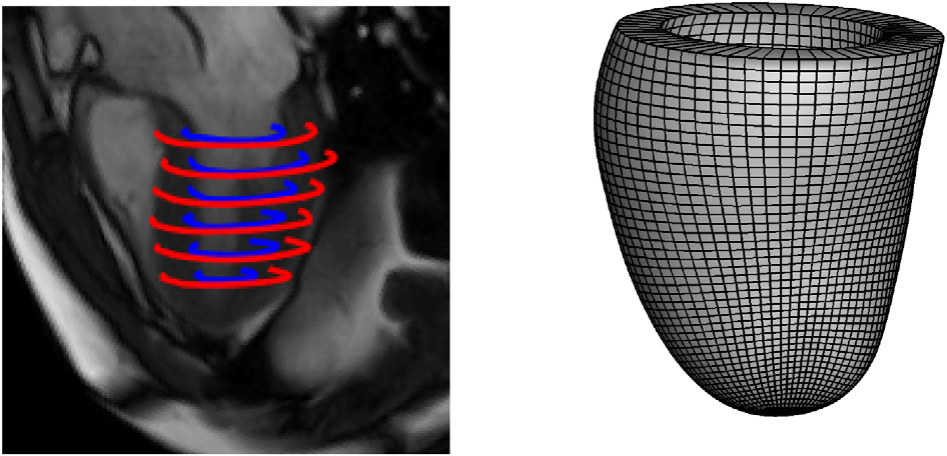
LV mesh reconstruction from a CMR image for a healthy volunteer. Left: segmented ventricular boundaries (blue: endocardium, red: epicardium) superimposed on a long axis CMR; right: the reconstructed LV geometry discretized with hexahedron elements

The boundary value problem (4) does not admit a closed‐form solution, hence numerical solutions are necessary in this context. The standard approach to solving biomechanical models is via the finite element method (FEM).^5^ In this work we use the nonlinear finite element software Abaqus FEA (Simulia, Providence, RI, USA) to solve the discretized cardio‐mechanics model. For a given parameter vector ϑ, the LV mesh, that is the discretized LV geometry at early diastole, and the desired end diastolic LV pressure, the Abaqus forward simulator will determine the deformed mesh at the end of diastole by balancing the internal and external mechanical energies under prescribed boundary conditions, see Figure 1. The deformed mesh is then used to extract segmental circumferential strains at corresponding segments defined in four short‐axial CMR cine images following clinical convention as specified by the American Heart Association (AHA),^42^ as well as to compute the cavity volume at the end of diastole. Figure 2 illustrates an example of the reconstructed LV geometry derived from in vivo cine images with manually defined wall boundaries. Details of the LV geometry reconstruction can be found in Gao et al.,^4^ including the definition of circumferential segmental strains.

The parameter vector ϑ describes the myocardial material properties and hence determines its mechanical responses through the HO model. Table 1 presents two sets of reference values of ϑ used in the literature. However, a subject‐specific value of ϑ can neither be directly observed nor measured in vivo. Thus, we are interested in inferring it using routinely‐available non‐invasive in vivo data obtained from CMR images. Figure 2, left, presents an instance of such a scan together with manually annotated LV boundaries for the epicardium (red) and endocardium (blue). To the best of our knowledge, Gao et al.^28^ were the first to address the issue of parameter inference in the HO model from in vivo clinical measurements (CMR scans). We discuss their algorithm, together with its updated version,^43^ in Section 2.1. Still, parameter estimation for the HO model using in vivo data remains a challenging problem due to the potential non‐identifiability of the constitutive parameters and their strong correlation,^28^, ^38^ as well as the high cost of solving the cardio‐mechanics model numerically.

**TABLE 1:** Reference parameters $\vartheta_{0}$ proposed in the literature

Work	*a*_0_ (kPa)	*b*_0_	$a_{f0}$ (kPa)	$b_{f0}$	$a_{s0}$ (kPa)	$b_{s0}$	$a_{\mathrm{fs}0}$ (kPa)	$b_{\mathrm{fs}0}$
Wang et al.^33^	0.236	10.810	20.037	14.154	3.724	5.164	0.411	11.300
Gao et al.^4^	0.18	2.6	3.34	2.73	0.69	1.11	0.31	2.58

*Note*: Wang et al.^33^ estimated their parameters based on ex vivo data, while Gao et al.^4^ used in vivo data.

### 2.1 Existing estimation methods

Gao et al.^28^ proposed a three step algorithm based on matching the responses from the forward simulator associated with the HO model with the in vivo measurements obtained from CMR scans (circumferential segmental strains and the end‐diastolic cavity volume). We refer to their approach as the HGO algorithm. The key idea behind the HGO algorithm is not estimating ϑ directly but via rescaling a reference value $\vartheta_{0}$, taken from an earlier study.^33^ Moreover, each step rescales a different subset of HO parameters.

Listing 1 presents steps of the HGO algorithm, where the scaling scalars $C_{a}$, $C_{b}$ and $C_{3}$ are defined in the following way. Scalars $C_{a}$ and $C_{b}$ are used to match the parameters in two parameter groups, $a^{\text{group}}=\left\{a,a_{f},a_{s},a_{\mathrm{fs}}\right\}$ and $b^{\text{group}}=\left\{b,b_{f},b_{s},b_{\mathrm{fs}}\right\}$, respectively, as

$$a^{\text{group}}=C_{a}a_{0}^{\text{group}},\;b^{\text{group}}=C_{b}b_{0}^{\text{group}},$$

while scalar $C_{3}$ matches a and $a_{\mathrm{fs}}$

$$a=C_{3}a^{\left(2\right)},\;a_{\mathrm{fs}}=C_{3}a_{\mathrm{fs}}^{\left(2\right)},$$

where $a^{\left(2\right)}$ and $a_{\mathrm{fs}}^{\left(2\right)}$ are values of a and $a_{\mathrm{fs}}$, respectively, optimised in the second step of the algorithm. The two objective functions matching the simulated values, depending on the constitutive parameter ϑ and geometry H, to the measurement are defined as follows

(5) $$f_{O1}\left(\vartheta ,H\right)={\left(V\left(\vartheta ,H\right)-V^{*}\right)}^{2}+\sum_{i=1}^{K}{\left(\varepsilon_{i}\left(\vartheta ,H\right)-\varepsilon_{i}^{*}\right)}^{2},$$

(6) $$f_{O2}\left(\vartheta ,H\right)=\frac{{\left(V\left(\vartheta ,H\right)-V^{*}\right)}^{2}}{V^{*}}+\sum_{i=1}^{K}{\left(\varepsilon_{i}\left(\vartheta ,H\right)-\varepsilon_{i}^{*}\right)}^{2},$$

where $V^{*}$ and $\varepsilon_{i}^{*}$, i=1,⋯,K=24 are the measurements of the LV cavity volume and 24 circumferential strains, respectively, Note the 24 circumferential strains are measured from 4 short‐axis slices from the base to the middle ventricle with 6 regions per slice. While $V\left(\vartheta ,H\right)$ and $\varepsilon_{i}\left(\vartheta ,H\right)$ are the corresponding values obtained from the forward simulator using the parameter vector ϑ and the LV geometry H.

#### 2.1.1 Updated algorithm

Gao et. al^43^ have developed an updated version of the original HGO algorithm,^28^ which we present in Algorithm 2. We can see that the main difference between the two versions is that the new one includes an additional step based on refining $C_{a}$ and $C_{b}$ with $f_{O2,\text{Klotz}}$, which we discuss in Section 2.2. In short, $f_{O2,\text{Klotz}}$ augments $f_{O2}$ with an additional term which, for the given parameters ϑ and LV geometry H, quantifies the discrepancy between the LV volume predicted for pressure of 30 mmHg and—since no in vivo data is observed at such a high pressure—the prediction from the so‐called Klotz‐curve,^35^ which we discuss in Section 2.2. This aims at inferring parameters which not only agree with the in vivo data observed at low pressures, but also give realistic prediction for high pressure scenarios. Another difference between Algorithms 1 and 2 can be observed in the last step: the updated version matches a and b while the original algorithm optimises a and $a_{\mathrm{fs}}$. There are two reasons for the latter modification. First, the sensitivity of $a_{\mathrm{fs}}$ is much lower compared to a and b ^28^ and it is more related to the shearing response, which does not directly link to the measured circumferential strains.^32^ Second, a and b are two highly sensitive parameters and directly related to the extracellular matrix response. Thus, in the final step a and b are updated to match the measured data.

ALGORITHM 1 **Original HGO algorithm of Gao et al.^28^**

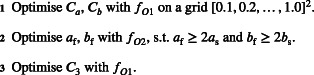

ALGORITHM 2 **Updated HGO algorithm of Gao et al.^43^**

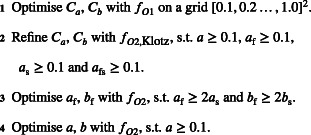

#### 2.1.2 Parameter reduction

The eight parameters in ϑ are highly correlated and not identifiable from limited data based on CMR scans.^28^ Therefore, we follow Davies et al.^38^ and use a parametrisation reduced to a 4–dimensional manifold

(7) $$\left.\begin{array}{ll} \;a=\theta_{1}a_{0}, & \;b=\theta_{1}b_{0},\\ \;a_{f}=\theta_{2}a_{f0}, & \;a_{s}=\theta_{2}a_{s0},\\ \;b_{f}=\theta_{3}b_{f0}, & \;b_{s}=\theta_{3}b_{s0},\\ a_{\mathrm{fs}}=\theta_{4}a_{\mathrm{fs}0}, & b_{\mathrm{fs}}=\theta_{4}b_{\mathrm{fs}0}, \end{array}\right\}$$

where $\theta ={\left(\theta_{1},\theta_{2},\theta_{3},\theta_{4}\right)}^{T}$ is the vector of scalings of the reference parameters $\vartheta_{0}={\left(a_{0},b_{0},a_{f0},b_{f0},a_{s0},b_{s0},a_{\mathrm{fs}0},b_{\mathrm{fs}0}\right)}^{T}$. Wang et al.^33^ proposed a popular reference parameter vector, used in several later studies.^28^, ^38^ However, those values were based on ex vivo data, making them potentially of limited relevance to our problem (inference based on in vivo data). Hence, we use different values for $\vartheta_{0}$ based on in vivo data population‐wide averages.^4^ The only exception is the implementation of the HGO algorithm,^28^ which originally uses the earlier reference values.^33^ We have implemented this algorithm using both reference parameter vectors.^4^, ^33^

### 2.2 Klotz‐curve

In vivo data are collected at physiologically normal (low) ventricular pressures. These pressures, however, are not recorded in healthy volunteers due to the invasive nature of the procedure. We assume a population‐based end‐diastolic (ED) pressure of 8 mmHg.^28^ Working with “low pressure data” implies we can only estimate those parameters of the HO model that describe myocardium properties at such low pressures. However, parameters leading to a similar low pressure behaviour (in terms of the implied stress–strain relationships) may result in a very different behaviour at high pressures, such as 30 mmHg. These high pressures are of interest as they may reveal LV stiffness with impaired relaxation, which characterises diastolic heart failure.^22^ Hence, we want to avoid that parameters which have been inferred from in vivo pressures exhibit physiologically unrealistic behaviour at high pressures.

However, high pressure in vivo volume measurements are not available, therefore we propose to predict them using the empirical law found by Klotz et al.,^35^ which we will refer to as the Klotz‐curve. This empirical relationship is valid for a wide range of hearts, including healthy and diseased (with congestive heart failure or left ventricular assist device support) human hearts as well animal (canine and rat) hearts. The normalised ED volume ${\tilde{V}}^{*}$ is defined as^35^

(8) $${\tilde{V}}^{*}=\frac{V^{*}-V_{0}}{V_{30}-V_{0}},$$

where $V^{*}$ is the measured unnormalised volume at $P^{*},V_{0}$ is the zero‐pressure volume called the load‐free volume. An empirical law based on population‐wide ex vivo data is then established^35^ relating the normalised LV volume to the corresponding measurement pressure $P^{*}$ with the specific form of

(9) $$P^{*}=A{\left({\tilde{V}}^{*}\right)}^{B},$$

where A and B are parameters. In our case $V^{*}$ is the in vivo measured volume at pressure $P^{*}=8$ mmHg. Combining (9) and (8) results in the following formula for the high‐pressure ED volume

(10) $$V_{30}^{\mathrm{Kl}}=V_{0}+\frac{V^{*}-V_{0}}{{\tilde{V}}^{*}}=V_{0}+\frac{V^{*}-V_{0}}{{\left(\frac{P^{*}}{A}\right)}^{1/B}},$$

where $V_{30}^{\mathrm{Kl}}$ denotes $V_{30}$ predicted using the Klotz‐curve. Note that the ED pressure‐volume relationship in (9) as well as the derived formula for the high‐pressure ED volume (10) are based on *normalised* volumes: volume normalisation was crucial to obtain a widely‐holding pressure‐volume relationship.^35^

Note that formula (10) depends on the load‐free volume $V_{0}$, which can only be measured ex vivo. With only in vivo measurements available, we need to make some assumption regarding how to estimate it. We use the early diastolic volume as a proxy for the load‐free volume, since it is known that the LV pressure is the lowest at early diastole.^28^, ^44^ Thus, we predict $V_{30}^{\mathrm{Kl}}$ using the approximated ${\hat{V}}_{0}$ and the in vivo measured volume $V^{*}$ at $P^{*}=8$ mmHg as

$${\hat{V}}_{30}^{\mathrm{Kl}}={\hat{V}}_{0}+\frac{V^{*}-{\hat{V}}_{0}}{{\left(P^{*}/\hat{A}\right)}^{1/\hat{B}}},$$

where $\hat{A}=27.78$ and $\hat{B}=2.76$ are the estimates.^35^

We use the Klotz‐curve predicted high‐pressure volume ${\hat{V}}_{30}^{\mathrm{Kl}}$ to add a high‐pressure penalty term to the objective function (6) as follows

(11) $$f_{O2,\text{Klotz}}\left(\vartheta ,H\right)={\left(\frac{V_{8}\left(\vartheta ,H\right)-V_{8}^{*}}{V_{8}^{*}}\right)}^{2}+\sum_{i=1}^{K}{\left(\varepsilon_{i}\left(\vartheta ,H\right)-\varepsilon_{i}^{*}\right)}^{2}+{\left(\frac{V_{30}\left(\vartheta ,H\right)-{\hat{V}}_{30}^{\mathrm{Kl}}}{{\hat{V}}_{30}^{\mathrm{Kl}}}\right)}^{2},$$

where $V_{8}$ and $V_{30}$ are LV cavity volumes from the forward simulator, at 8 mmHg and 30 mmHg, respectively; $\varepsilon_{i}$, i=1,⋯,K=24, are regional circumferential strains from the forward simulator at 8 mmHg; $V_{8}^{*}$ is the in vivo measured LV cavity volume (at the normal pressure of 8 mmHg).

## 3 BAYESIAN OPTIMISATION

Suppose we are interested in finding the global optimum of a smooth function $f\left(x\right):{\mathbb{R}}^{d}⊃X\to \mathbb{R}$. We will refer to f as the objective or target function and without loss of generality assume it is a loss function to be minimised. Further, suppose computing f at any given x is expensive and/or time‐consuming so that we want to find the optimiser $x^{*}=\arg\min_{x}f\left(x\right)$ in as few steps as possible. BO proceeds in an iterative fashion, at each iteration t selecting an input $x_{t}$ and querying the black‐box function $y_{t}=f\left(x_{t}\right)$. Input selection is done by optimising the so‐called acquisition function (AF), which is based on an approximation of f with a surrogate and models the desired trade‐off between *exploration* (searching for new promising regions) and *exploitation* (increasing the resolution around beneficial regions already discovered).

### 3.1 Gaussian process surrogates

Typically, the AF is based on a *surrogate model* of the objective function f, with a popular choice for such a surrogate being Gaussian processes (GPs). As opposed to alternative nonlinear methods, such as for example splines and neural networks, GPs allow exact interpolation rather than regression, which is what is conceptually required for deterministic objective functions, such as the one based on a deterministic cardiomechanic model. However, due to technical reasons discussed below, restating the interpolation problem as a regression problems is more practical, so that below we focus on the GP regression setting.

In BO GP regression is trained on a sequentially expanding set of inputs $X_{t}={\left[x_{j}\right]}_{j=1}^{t}$ and outputs $Y_{t}={\left[y_{j}\right]}_{j=1}^{t}$. We refer to Rasmussen and Williams^8^ for an extensive treatment of GPs. GP regression is a nonparametric model in which a GP prior is put over the unknown function of interest to represent our initial beliefs about its smoothness. The target values at points $x_{j}$, j=1,…,t observed up to iteration *t* are given by

(12) $$y_{j}=f\left(x_{j}\right)+\varepsilon_{j},\;\varepsilon_{j}\overset{\mathrm{iid}}{∼}N\left(0,\sigma^{2}\right),$$

where $N\left(\mu ,\sigma^{2}\right)$ denotes the Gaussian distribution with mean μ and variance $\sigma^{2}$ (and *iid* stands for independently and identically distributed). Specification (12) is a general one, allowing for noisy observations. Our forward simulator of interest is deterministic, so mathematically there is no noise ($\sigma^{2}=0$). However, including a non‐trivial noise term $\varepsilon_{j}$ is still convenient for at least two reasons. First, in practice there may be numerical noise, due to the finite precision of the numerical solution of the forward problem. Second, when working with GPs it is a common practice to allow for a small *jitter* (or nugget) term (e.g. 10^−9^), which is added to the diagonal of the training covariance matrix for numerical stability of the matrix inversion. Effectively, jitter is imposed noise, which slightly dilutes the informativeness of our deterministic data.

In (12) the latent values $f\left(x\right)$ follow a GP

$$f\left(x\right)∼\mathrm{GP}\left(\mu \left(x\right),k\left(x,x\prime \right)\right),$$

where

$$\mu \left(x\right)=E\left[f\left(x\right)\right],\;k\left(x,x\prime \right)=E\left[\left(f\left(x\right)-\mu \left(x\right)\right)\left(f\left(x\prime \right)-\mu \left(x\prime \right)\right)\right]$$

are the prior mean and the covariance function (kernel) of the process f, respectively. Hence, it is assumed that the responses $y_{j}$ are conditionally independent given the latent values $f\left(x_{j}\right)$. Below we adopt a standard assumption that $\mu \left(x\right)=0$ so the latent process $f\left(x\right)$ is fully specified by its kernel function.

The kernel can be specified in many different ways, including numerous standard functional forms.^8^ We consider the Matérn 3/2 kernel with automatic relevance determination (ARD) given as

$$k_{0}\left(x,x\prime \right)=\sigma_{k}^{2}\left(1+\sqrt{3}r\right)\exp\left(-\sqrt{3}r\right),\;r={\left(\sum_{i=1}^{d}\frac{{\left(x_{i}-x_{i}^{\prime }\right)}^{2}}{l_{i}^{2}}\right)}^{1/2},$$

where $l_{i},i=1,\ldots ,d$ is the length scale parameter for the *i*th input variable and ARD refers to this allowing for a different length scale for each dimension. Thus our GP regression model in (12) is parametrised by a vector of hyperparameters $\phi ={\left(\sigma^{2},\sigma_{k}^{2},l_{1},\ldots ,l_{d}\right)}^{T}$, so that we consider the observation noise variance $\sigma^{2}$ a hyperparameter.^8^ The Matérn 3/2 kernel produces continuous trajectories with continuous derivatives at the same time providing a considerable degree of roughness (it is once differentiable) allowing us to model functions with cliffs and plateaus. Although this choice of kernel rules out standard second‐order optimisation methods, like quasi‐Newton, we note that second‐order methods are of limited use in the presence of extensive multimodality (which is a typical problem when optimising AFs, see Section 3.2.1). Moreover, based on our experimentation this low‐level Matérn class leads to numerically more stable covariance matrix inversion than a higher‐level Matérn class.

For the collected inputs $X_{t}$ we obtain the GP prior over function values $p\left(f_{t}|X_{t},\phi \right)=N\left(f_{t}|0,K_{t}\right),$ with $f_{t}={\left[f\left(x_{j}\right)\right]}_{j=1}^{t}$ and $K_{t}=k\left(X_{t},X_{t}\right)$. The likelihood is given by $p\left(Y_{t}|f\right)=N\left(Y_{t}|f_{t},\sigma^{2}I\right)$, where I is the identity matrix, and marginalising over the latent variables $f_{t}$ gives the formula for the marginal likelihood

$$p\left(Y_{t}|X_{t},\phi \right)=N\left(Y_{t}|0,K_{t}+\sigma^{2}I\right).$$

Under the adopted Gaussian observation model (12) the conditional posterior distribution of the latent variables also becomes Gaussian, with the following form

$$p\left(f_{t}|Y_{t},X_{t},\phi \right)=N\left(K_{t}{\left(K_{t}+\sigma^{2}I\right)}^{-1}Y_{t},K_{t}-K_{t}{\left(K_{t}+\sigma^{2}I\right)}^{-1}K_{t}\right).$$

### 3.2 Acquisition function

The performance of BO algorithms depends on the particular choice of AF. Various AFs have been proposed in the literature,^6^ but the Expected Improvement^10^ criterion remains one of the most popular choices. EI has gained popularity as it is conceptually simple (as opposed to e.g. information theoretic AFs), it admits a closed form expression for GP surrogates and performs well for a range of problems.^11^ As its name suggests, EI quantifies the expected improvement, that is the amount of improvement over the current *incumbent* $f^{*}$, that is the best (lowest) value of f found so far, weighted by the probability of it occurring. EI is defined as

$$\mathrm{EI}\left(x\right)=E_{p\left(y|x,D\right)}\left[\min\left(f^{*}-f\left(x\right),0\right)\right],$$

where D denotes the set of inputs and outputs recorded so far and $p\left(y|x,D\right)$ is the predictive posterior output distribution given the chosen evaluation point **x** and the previously observed D. With a GP surrogate $\mathrm{GP}\left(\mu \left(x\right),k\left(x\right)\right)$ EI can be expressed^6^, ^10^ as

$$\mathrm{EI}\left(x\right)=\left(f^{*}-\mu \left(x\right)\right)\Phi \left(z\right)+\sqrt{k\left(x\right)}\phi \left(z\right),$$

where $z=\left(f^{*}-\mu \left(x\right)\right)/\sqrt{k\left(x\right)}$ and Φ and ϕ are the CDF and PDF of the standard normal distribution, respectively. The EI is made up of two terms. The first term is increased by decreasing the predictive mean $\mu \left(x\right)$, the second term is increased by increasing the predictive uncertainty $k\left(x\right)$. This shows how EI automatically balances exploitation and exploration.

#### 3.2.1 Inner optimisation

BO selects the next query point based on maximising the AF, which is often referred to as *inner optimisation* as it is within the *outer optimisation* (optimising the unknown objective function f) of the BO algorithm. Even though inner optimisation does not require any computationally expensive forward simulations, it can be a challenging task.^7^ This is often due to the observed multimodality of EI, which typically is picked around several points and flat between them. A popular approach to overcome this problem is the multi‐starts method,^45^ which performs standard numerical optimisation using for example quasi‐Newton methods such as BFGS multiple times, from different initial points. However, a pilot study we conducted revealed that the multi‐starts method performs poorly in our application, irrespective of a particular kernel choice (we tested several standard kernels, such as squared exponential, Matérn 5/2, Matérn 3/2 and neural network), often indicating suboptimal points and thus leading to unstable BO runs. That was the case even with an atypically large number of restarts (set to 1000 and drawn from a Latin hypercube design).

For these reasons we use a different approach based on a global optimisation algorithm called OQNLP^46^ (or Global Search in its implementation in MATLAB's Global Optimisation toolbox that we use), which led to very stable results. In short, the OQNLP is a heuristic algorithm also based on multiple starting points. Those starting points, however, are not randomly (or quasi‐randomly) selected, but rather calculated from the scatter search algorithm.^47^ They are then scored based on the corresponding value of the objective function and, if present, constraint satisfaction. Next, gradient‐based nonlinear programming (NLP) solvers are run from the starting point with the highest score value. Thereafter, the remaining points are analysed and those that are unlikely to improve the best local minimum found so far are rejected (which is different to the standard multistart approach in which solvers are run for all the initial points). The filtering analysis takes into account the corresponding value of the objective function, constraint violations and basins of attraction. The latter means that the points in the same basins of attraction as previously analysed points are excluded from further NLP optimisation. This feature, that is analysis of basis of attraction, contributes to the efficiency of the OQNLP algorithm as it allows for discarding points which would converge to the already found local optima.

#### 3.2.2 Unknown constraints

It may happen during BO iterations that the proposed point, in spite of maximising the AF based on the current surrogate, will violate the biomechanical assumptions behind the HO law (we refer the reader to Appendix F) for an analysis of simulator crashes. Such a situation will demonstrate itself as a crash of the associated forward simulator, which is inefficient as some computations will be carried out in vain. The problem of a simulator failing to terminate or crashing was addressed in the literature^36^, ^37^ by weighting EI by the probability of the constraint being satisfied

$${\mathrm{EI}}_{\mathrm{con}}\left(x\right)=\mathrm{EI}\left(x\right)\mathbb{P}\left(x\in C\right),$$

where $\mathbb{P}\left(x\in C\right)$ is the probability of x being a valid point not leading to a crash of the forward simulator.

To model the probability of the unknown constraint being satisfied we use a GP classifier with a probit likelihood^36^ with approximate inference carried out using expectation propagation.^48^ Similarly as for the regression model, we use the Matérn 3/2 kernel. A probit likelihood is a conceptually sound approach, however it means that we lose the analytic tractability of the standard EI function as for non‐Gaussian likelihoods approximate inference methods (such as Laplace approximation,^49^ variational inference,^50^ expectation propagation^48^) are necessary.^8^ A simpler though heuristic classifier could be provided by the so‐called label regression^51^ (LR). LR is an ordinary linear regression model fitted to explain a binary outcome variable and its advantage is that $\mathbb{P}\left(x\in C\right)$ can be computed explicitly, yet based on a suboptimal probability model (a Gaussian, rather than a logit or probit model). LR has been successfully employed to account for hidden constrains in the context of BO applied to pulmonary circulation.^52^ However, in our preliminary study we found that in our application LR led to unstable results, generally performing poorly.

We note that the probability of a crash happening during a BO run is LV geometry and material parameter dependent. For the investigated LV geometries crashes are not very frequent, see Sections 4.3 and 4.4, however, we note that the reported numbers of crashes, if nonzero, are already based on training a classifier. In fact, our pilot study revealed that not allowing for any learning from past unsuccessful simulations can lead to repeatedly querying very similar “crash points”, resulting in much higher numbers of crashes.

#### 3.2.3 Partial error surrogates

Notice that both target functions, $f_{O2}$ and $f_{O2,\text{Klotz}}$, are sums of individual squared error terms. Thus, instead of developing a surrogate for the target as a whole we propose to construct a surrogate for each error term separately. In the context of emulation^38^ a similar approach led to a substantial improvement in accuracy by emulating the vector of outputs from the cardiac mechanic model. That approach is efficient in the context of emulation when the developed emulator is aimed to be used for a wide range of different subjects. However, in our context using surrogate functions for the outputs would require us to *approximate* EI, as implied by the predictive means from individual output surrogates. Considering surrogates for the individual error terms instead allows us to obtain a closed form expression for EI. Note that considering partial error surrogates instead of output surrogates does not cause an efficiency loss as in our case optimisation is carried out for subject‐specific measurements, known before running computations (which is different than for emulators).

We refer to the proposed approach as *partial error surrogates*.

Let

(13) $$f\left(\vartheta ,H\right)=\sum_{i=1}^{K^{*}}f^{\left(i\right)}\left(\vartheta ,H\right),$$

where f is either $f_{O2}$ in (6) so that $K^{*}$ = *K* + 1 = 25, or $f_{O2,\text{Klotz}}$ in (11) so that $K^{*}$ = *K* + 2 = 26, and each $f^{\left(i\right)}$ refers to the squared error terms on the right‐hand side in those formulae. We assume that $f^{\left(i\right)}$s are mutually independent and model each $f^{\left(i\right)}$ using a GP regression parametrised by hyperparameters $\phi^{\left(i\right)}$. Allowing for correlated $f^{\left(i\right)}$s would require us to use computationally much more expensive multi‐output GPs,^53^, ^54^, ^55^ which would increase the computational overhead of each BO iteration, diminishing the posited “cheapness” of the surrogate compared to the forward simulator. Each partial GP regression has the conditional posterior mean $\mu_{t}^{\left(i\right)}\left(x\right)$ and covariance $k_{t}^{\left(i\right)}\left(x\right)$

$$f_{t}^{\left(i\right)}|Y_{t}^{\left(i\right)},X_{t},\phi^{\left(i\right)}∼\mathrm{GP}\left(\mu_{t}^{\left(i\right)}\left(x\right),k_{t}^{\left(i\right)}\left(x\right)\right),$$

where $f_{t}^{\left(i\right)}={\left[f^{\left(i\right)}\left(x_{j}\right)\right]}_{j=1}^{t}$ are the latent variables for the ith output from the cardiomechanic model and $Y_{t}^{\left(i\right)}={\left[y_{j}^{\left(i\right)}\right]}_{j=1}^{t}$ are the observed values for the ith output. Then the conditional posterior mean $\mu_{t}$ of the full target f is just the sum of conditional posterior means $\mu_{t}^{\left(i\right)}$ from the partial surrogates and the conditional posterior variance $k_{t}$ is the sum of the partial variances $k_{t}^{\left(i\right)}$:

$$f_{t}|Y_{t}^{\left(1\right)},\ldots ,Y_{t}^{\left(K^{*}\right)},X_{t},\phi^{\left(1\right)},\ldots ,\phi^{\left(K^{*}\right)}∼\mathrm{GP}\left(\mu_{t}\left(x\right),k_{t}\left(x\right)\right),$$

with

$$\mu_{t}\left(x\right)=\sum_{i=1}^{K^{*}}\mu_{t}^{\left(i\right)}\;\text{and}\;k_{t}\left(x\right)=\sum_{i=1}^{K^{*}}k_{t}^{\left(i\right)}.$$

#### 3.2.4 Standardisation

Standardising inputs and outputs is a common practice when working with GPs, aimed to provide numerical stability of the computations. In our case input standardisation is not an issue as all the partial surrogates use the same inputs. Predictions for the outputs, however, enter the formulae for the conditional posterior distribution (14) so we need to “de‐standardise” the predictions to obtain correct partial moments. Let ${\tilde{Y}}_{t}^{\left(i\right)}$ denote the ith, $i=1,\ldots ,\;K^{*}$, standardised partial squared error

$${\tilde{Y}}_{t}^{\left(i\right)}=\frac{Y_{t}^{\left(i\right)}-m_{0}^{\left(i\right)}}{\sigma_{0}^{\left(i\right)}},$$

where $m_{0}^{\left(i\right)}$ and $\sigma_{0}^{\left(i\right)}$ are the mean and standard deviation, respectively, used to standardise the ith partial squared errors (e.g. calculated on the initial design). The reverse transformation is given as

$$Y_{t}^{\left(i\right)}=g\left({\tilde{Y}}_{t}^{\left(i\right)}\right)=m_{0}^{\left(i\right)}+\sigma_{0}^{\left(i\right)}{\tilde{Y}}_{t}^{\left(i\right)},$$

so if the ith GP surrogate predicts $N\left({\tilde{\mu }}_{t}^{\left(i\right)},{\tilde{k}}_{t}^{\left(i\right)}\right)$ for the standardised ith partial squared error, this corresponds to predicting $N\left(g\left({\tilde{\mu }}_{t}^{\left(i\right)}\right),{\left(\sigma_{0}^{\left(i\right)}\right)}^{2}\;{\tilde{k}}_{t}^{\left(i\right)}\right)$ for the original ith partial squared error. Then the moments (14) used in the acquisition function are given by

$$\mu_{t}\left(x\right)=\sum_{i=1}^{K^{*}}\left(m_{0}^{\left(i\right)}+\sigma_{0}^{\left(i\right)}{\tilde{\mu }}_{t}^{\left(i\right)}\right)\;\text{and}\;k_{t}\left(x\right)=\sum_{i=1}^{K^{*}}{\left(\sigma_{0}^{\left(i\right)}\right)}^{2}\;{\tilde{k}}_{t}^{\left(i\right)}.$$

### 3.3 Emulator for high‐pressure volume

Computing $f_{O2,\text{Klotz}}$ in (11) requires running the forward simulator twice, at 8 mmHg and 30 mmHg, which requires double computing time/power. Each of these runs may be unsuccessful, that is may lead to a crash. However, parameters leading to crashes may differ between the 8 and 30 mmHg cases. Hence, we would need two classifiers, one for the 8 mmHg case and one for the 30 mmHg case, and we could only update the GP surrogate for $f_{O2,\text{Klotz}}$ when both cases terminate successfully.

To overcome these problems and to reduce the computational costs we propose to obtain the value for $V_{30}$ using an emulator rather than by running the forward simulator. Emulators of cardiac mechanics models often include constitutive parameters as inputs.^38^ However, the outputs from the forward simulator depend not only on the constitutive parameters but also on the LV geometry. Since we want the $V_{30}$ emulator to approximate $V_{30}$ for a broad range of LV geometries (as opposed to developing a geometry‐specific emulator^38^) we want to include some information about the LV geometry as input. However, we cannot use the LV mesh directly as it is a high‐dimensional (with thousands of coordinates) object and such a dimension is prohibitively high for any emulator. Our preliminary experimentation revealed that accurately predicting only the LV volume at 30 mmHg, and not the strains, does not require much details about a subject's specific shape of the LV and can be done based on a single “summary statistic” of the LV mesh—an approximation ${\hat{V}}_{0}$ to the load‐free LV cavity volume $V_{0}$ (see Section 2.2)—without a substantial loss in the accuracy of the emulator. We note that if we were also after emulating circumferential strains at 30 mmHg, accounting for more subtle features of the LV geometry would be necessary. To sum up, we train the $V_{30}$ emulator on the scaling parameters θ and the load‐free volume ${\hat{V}}_{0}$, in which we approximate the true function $V_{30}\left(\theta ,H\right)$ by $V_{30}\left(\theta ,V_{0}\right)$.

### 3.4 Final algorithm

Listings 3 and 4 summarise the initialisation and iterations, respectively, of the BO algorithm with the constraint‐weighted EI, Klotz‐curve objective (11), partial error surrogates and $V_{30}$ emulator.

ALGORITHM 3 **Initialisation of Bayesian optimisation with partial error surrogates**

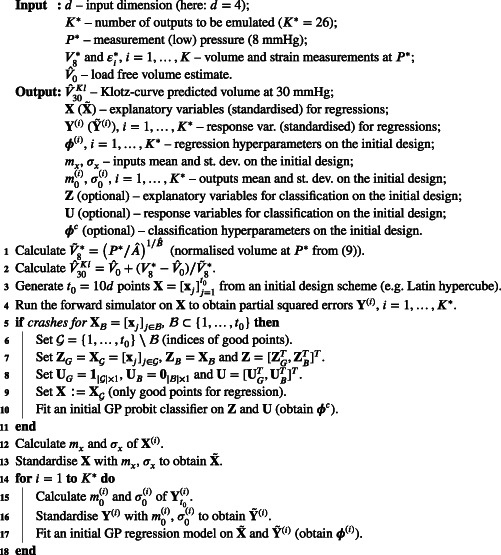

ALGORITHM 4 **Iterations of Bayesian optimisation with partial error surrogates**

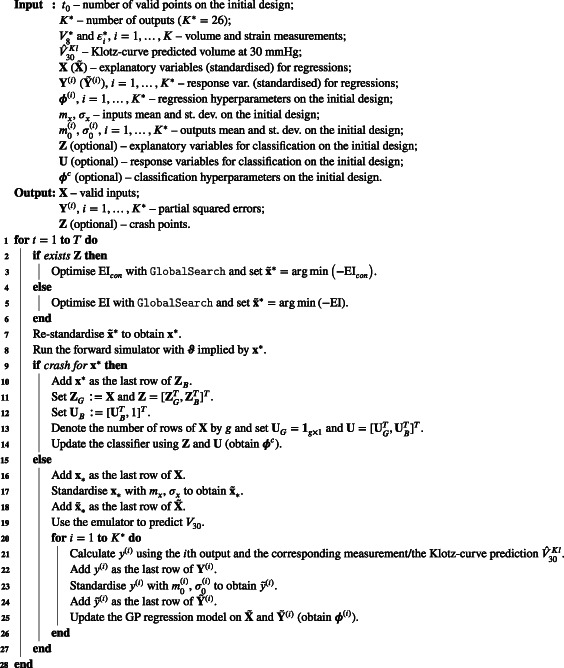

## 4 APPLICATIONS

This section presents three applications of BO to the problem of parameter inference in the HO model of the LV. We start with comparing BO *without* the Klotz‐curve prior with the original HGO algorithm,^28^ which we refer to as the *basic study*. We demonstrate that BO attains a similar or a lower value of the objective function *f*_*O*2_ in considerably fewer iterations (Section 4.3). Next, in the *Klotz‐curve study* we compare the versions of both algorithms that include the Klotz‐curve (Section 4.4). We show that also in this case BO typically outperforms the multi‐step algorithm, even though the updated HGO algorithm^43^ works better than its original version. In both these studies we consider four healthy volunteers (HVs), same in both applications, which we label HV A, HV B, HV C and HV D. Finally, in Appendix C we investigate theoretical accuracy of the proposed method by means of a synthetic data study based on HV A from the basic study.

### 4.1 Set‐up

In all our applications we compare two variants of BO, with target and partial error surrogates (13) (see Section 3.1 and 3.2.3). We use 500 iterations for BO, which are run after 40 iterations corresponding to an initial design obtained with Latin hypercube sampling,^56^ in which we follow a commonly followed rule‐of‐thumb^10^ to use 10*d* points to initialise optimisation (for a *d*‐dimensional problem). We use a recent value for $\vartheta_{0}$ (defined below (7)) based on in vivo data^4^ for both BO and the HGO algorithm, except the original HGO algorithm in the *basic study* for which we use the reference value from Wang et al.,^33^ originally used in the HGO algorithm.

### 4.2 Evaluation

In our result analysis we focus on three aspects. First, we consider the value of the objective function, as this is what both algorithms directly target. In the *basic study* this is *f*_*O*2_ given in (6), in the two later studies this is $f_{O2,\text{Klotz}}$ given in (11). Visually, we analyse the convergence of the *incumbent*, that is the best value of the objective function recorded so far, which is standard in the BO literature.^6^, ^37^ The convergence is with respect to the invocations of the forward simulator, which are the main computational burden and which we want to limit. The physical computing time is a less reliable measure of computing costs as it necessarily depends on the machine used, number of task performed in parallel, etc., and does not generalise to other mechanistic models with different computational costs of the forward problem. Note that the HGO algorithm is based on optimisation solvers implemented as “black‐boxes” (such as the trust‐region‐reflective algorithm or the sequential quadratic programming algorithm in MATLAB), so that tracking the incumbent over the individual iterations is not directly available. Moreover, each step in HGO has its own stopping criterion determining the number of iterations carried out in each step. Therefore, for HGO we just report the final value of the objective $y_{\min}^{\mathrm{HGO}}$ and the corresponding number of iterations $i_{\min}^{\mathrm{HGO}}$ (defined as the sum of iterations performed in all steps).

Quantitatively, we compare BO and HGO based on $\left(y_{\min},i_{\min}\right)$ and $\left(y\left(i_{\min}^{\mathrm{HGO}}\right),i\left(y_{\min}^{\mathrm{HGO}}\right)\right)$. The former pair is concerned with the lowest value attained by BO over all 540 iterations $y_{\min}$ and the number of iterations completed to obtain $y_{\min}$ denoted $i_{\min}$. These values are compared with their HGO counterparts, $y_{\min}^{\mathrm{HGO}}$ and $i_{\min}^{\mathrm{HGO}}$. The latter pair relates BO iterations to the final result from the HGO algorithm: $y\left(i_{\min}^{\mathrm{HGO}}\right)$ is the value of a BO run after the number of iterations used by the HGO algorithm $i_{\min}^{\mathrm{HGO}}$, while $i\left(y_{\min}^{\mathrm{HGO}}\right)$ is the number of iterations required to achieve at least as good a performance as the HGO algorithm. If after 540 iterations the value of the objective obtained with BO is still higher than the final value from the HGO algorithm we set $i\left(y_{\min}^{\mathrm{HGO}}\right)=\emptyset$. Table 2 summarises the notation used in both empirical studies.

**TABLE 2:** Notation used in the empirical studies

Symbol	Meaning
$y_{\min}$	The lowest value of the $f_{O2,\text{Klotz}}$ objective function
$y_{\min}^{S}$	$y_{\min}$ but with the Klotz error computed using the simulator (not emulator)
$i_{\min}$	Number of iterations leading to $y_{\min}$ ($y\left(i_{\min}\right)=y_{\min}$)
$y\left(i_{\min}^{\mathrm{HGO}}\right)$	Value of the objective function after the number of iterations used by HGO
$y^{S}\left(i_{\min}^{\mathrm{HGO}}\right)$	$y\left(i_{\min}^{\mathrm{HGO}}\right)$ but with the Klotz error computed using the simulator (not emulator)
$i\left(y_{\min}^{\mathrm{HGO}}\right)$	Number of iterations required to achieve at least as good a performance as the HGO algorithm (*∅* if that did not happen within 540 iterations)
$V_{30}$	LV volumes at 30 mmHg, computed with emulator or simulator at $y_{\min}$ or $y\left(i_{\min}^{\mathrm{HGO}}\right)$:
	$E\left(y_{\min}\right)$ when computed for $y_{\min}$ with the emulator
	$S\left(y_{\min}\right)$ when computed for $y_{\min}$ with the simulator
	$E\left(i_{\min}^{\mathrm{HGO}}\right)$ when computed for $y\left(i_{\min}^{\mathrm{HGO}}\right)$ with the emulator
	$S\left(i_{\min}^{\mathrm{HGO}}\right)$ when computed for $y\left(i_{\min}^{\mathrm{HGO}}\right)$ with the simulator
#Cr.	Total number of crashes of the forward simulator

Second, we analyse the *stress‐stretch curves* corresponding to the final parameter estimates. We consider two virtual uni‐axial stretch experiments, one is along the myofibre direction (myocytes), and the other one is along the sheet direction (transmurally), often adopted in the literature for characterising nonlinear myocardial property.^28^, ^39^, ^57^ Stretch‐stress curves present the relationship between stress and stretch along two axes in the myocardium, along the myocyte and along the sheet direction. They provide insights into the predicted stiffness of the myocardium, with higher values of stress for a given stretch value indicating a stiffer material. They are of interest to cardiac mechanics specialist and clinicians as the parameters of the HO model cannot always be uniquely identified using experimental data^28^ and further due to the nonlinear myocardium response. The latter makes the differences in the predicted parameters not fully informative about the implied output differences. For the reproducibility of stretch‐stress curves, we report the underlying parameters in Appendix A.

**FIGURE 3:**
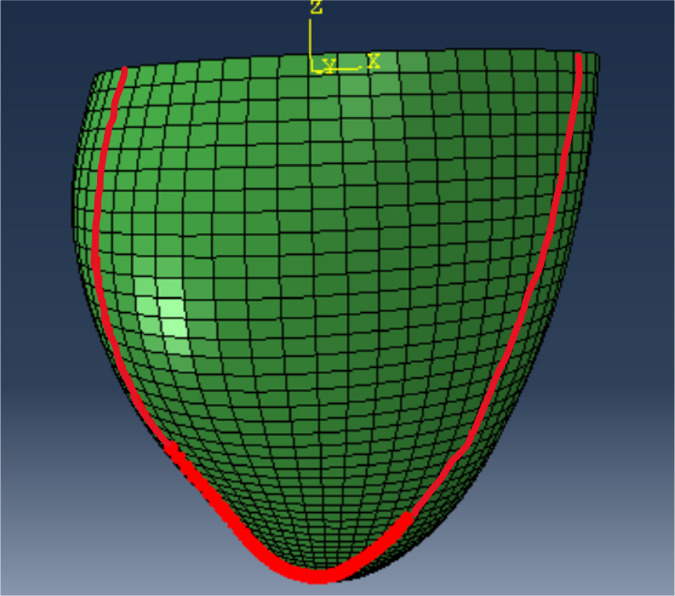
A defined reference line in the epicardial surface for illustrating the deformed LV shape^58^

Third, we consider deformations of the 3D LV geometry implied by the final parameter estimates. The underlying rationale is that uni‐axial stretch‐stress curves provide only a simplified test and do not capture the full picture of the deformation of the whole LV geometry. Following a cardiac mechanics benchmark paper,^58^ we illustrate the deformation of a line in the epicardial surface (see Figure 3), with a specific focus on two regions: the inflexion point near the middle ventricle and the apical region. In this section we discuss deformations at the standard pressure of 8 mmHg, while Appendix D provides additional figures related to deformation at 30 mmHg. The latter, much higher pressure may be useful for comparing estimates as it leads to exaggerated differences in the implied deformations because of the intrinsic nonlinearity in the HO model.

Both BO and HGO provide point estimates. Given an increasing awareness in the cardiac modelling community of the importance of uncertainty quantification and sensitivity analysis for model parameter estimates,^59^, ^60^, ^61^ we discuss in Appendix E how to quantify uncertainty of BO estimates in a straightforward way. We then illustrate one of the available methods, the residual bootstrap approach, in a computational experiment.

### 4.3 Basic study

Gao et al.^28^ have established a benchmark for parameter inference in the HO model. Their algorithm (see Listing 1) does not involve the Klotz‐curve prior discussed in Section 2.2, hence for comparability we first consider a version of BO without the Klotz‐curve prior, with the objective function given by *f*_*O*2_ from (6).

We appreciate that the HGO algorithm runs with respect to different parameters than the proposed BO framework and that the number of parameters used by both algorithms is different (in particular, in HGO the number of parameters with respect to which the optimisation is carried out may vary between the algorithm steps). This may raise concerns about the fairness of the BO–HGO comparison. Therefore, in Appendix G we present a comparison of BO against an established iterative optimisation scheme in the same 4‐dimensional parameter space and demonstrate the superiority of BO also in this context.

**FIGURE 4:**
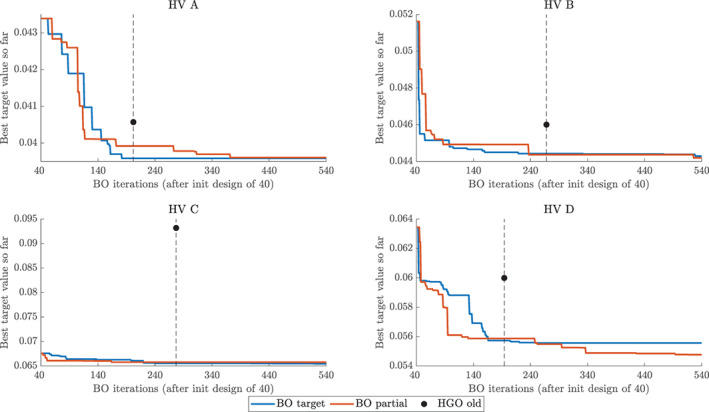
Basic study: convergence of the objective function $f_{O2}$ for Bayesian optimisation and the original HGO algorithm for four LV geometries (HV A, HV B, HV C, HV D). Horizontal axis: Bayesian optimisation iterations starting from 40 iterations for the initial design. Vertical axis: best value of the objective function $f_{O2}$ recorded so far. Black dot and horizontal dashed line: the final value of the objective function $f_{O2}$ for the HGO algorithm and the associated number of iterations. Bayesian optimisation with a target surrogate (target) and a partial error surrogate (partial) together with the old version of the HGO algorithm (HGO old)

Figure 4 presents the convergence of the *incumbent* for the four considered HVs. We observe that BO converges very quickly, typically in around 200 iterations after the initial design, so that the graph stays flat over the remaining iterations. The HGO algorithm typically requires more iterations to terminate. Moreover, in all the cases BO converges to a lower value of the objective function than HGO. In the most extreme case of HV C, the lowest value found on the BO initial design is lower than the final value for HGO. The two variants of BO, with target and partial error surrogates (13) (see Section 3.1 and 3.2.3) generally perform comparably, except for HV D, for which BO with a partial error surrogate converges faster and to a better point than BO with a target surrogate.

The main quantitative insights from Figure 4 are summarised in Table 3. We can see that $y_{\min}$ obtained with BO are always lower than their HGO counterparts. However, in most cases (except HV A and BO with a target surrogate) they are obtained using more iterations than HGO. A fairer comparison is hence provided by $y_{\min}^{\mathrm{HGO}}$. Even after less iterations BO still provides lower values of the objective function. This better performance of BO can be alternatively assessed using $i\left(y_{\min}^{\mathrm{HGO}}\right)$ which shows that for HV B, HV C and HV D we record at least as good performance of BO as for HGO using 4–7 times less iterations.

**TABLE 3:** Basic study: convergence of the objective function for Bayesian optimisation and the original HGO algorithm for four LV geometries (HV A, HV B, HV C, HV D)

HV	Algorithm	$y_{\min}$	$i_{\min}$	$y\left(i_{\min}^{\mathrm{HGO}}\right)$	$i\left(y_{\min}^{\mathrm{HGO}}\right)$	#Cr.
HV02	BO targ.	0.0396	181	0.0396	129	1
	BO part.	0.0396	371	0.0399	113	1
	HGO old	0.0406	201	0.0406	201	0
HV B	BO targ.	0.0443	528	0.0444	45	0
	BO part.	0.0442	525	0.0444	56	6
	HGO old	0.0460	267	0.0460	267	0
HV C	BO targ.	0.0655	518	0.0656	40	3
	BO part.	0.0658	499	0.0658	40	0
	HGO old	0.0932	276	0.0932	276	0
HV D	BO targ.	0.0556	247	0.0557	46	7
	BO part.	0.0548	516	0.0559	47	0
	HGO old	0.0600	193	0.0600	193	0

*Note*: Bayesian optimisation with a target surrogate (targ.) and a partial error surrogate (part.) together with the old version of the HGO algorithm (HGO old).

**FIGURE 5:**
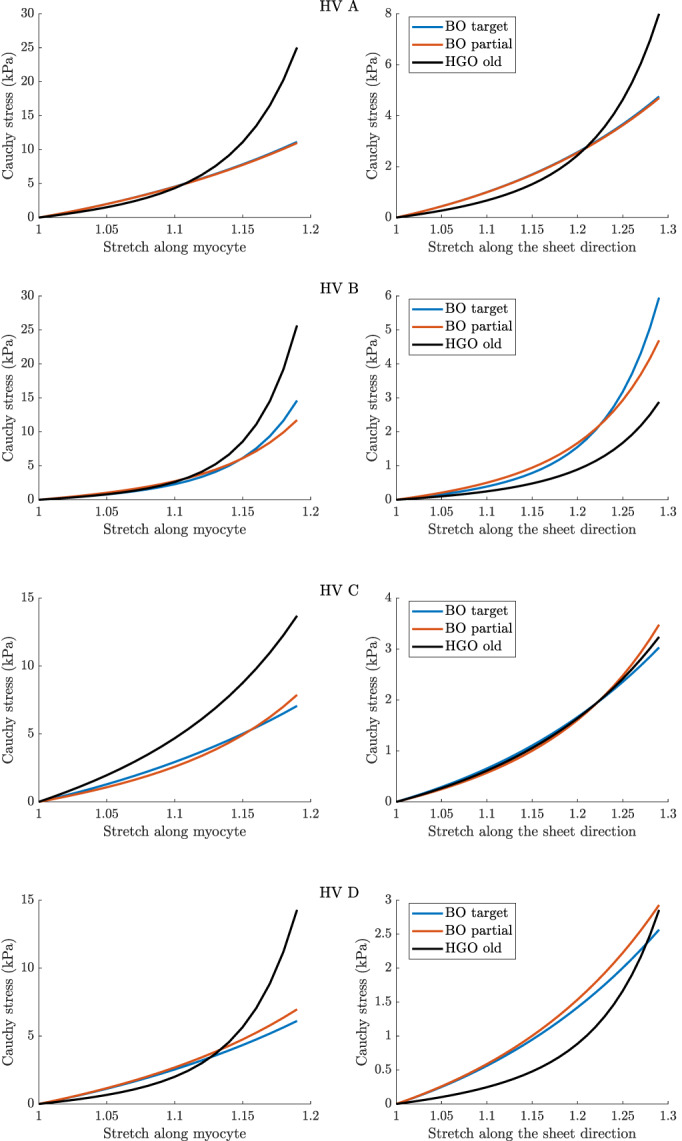
Basic study: stretch‐stress curves for four LV geometries (HV A, HV B, HV C, HV D). Left: responses to stretches along the myocyte direction $f_{0}$, right: responses to stretches along the sheet direction $s_{0}$ (see (2)). Bayesian optimisation with a target surrogate (target) and a partial error surrogate (partial) together with the old version of the HGO algorithm (HGO old)

**FIGURE 6:**
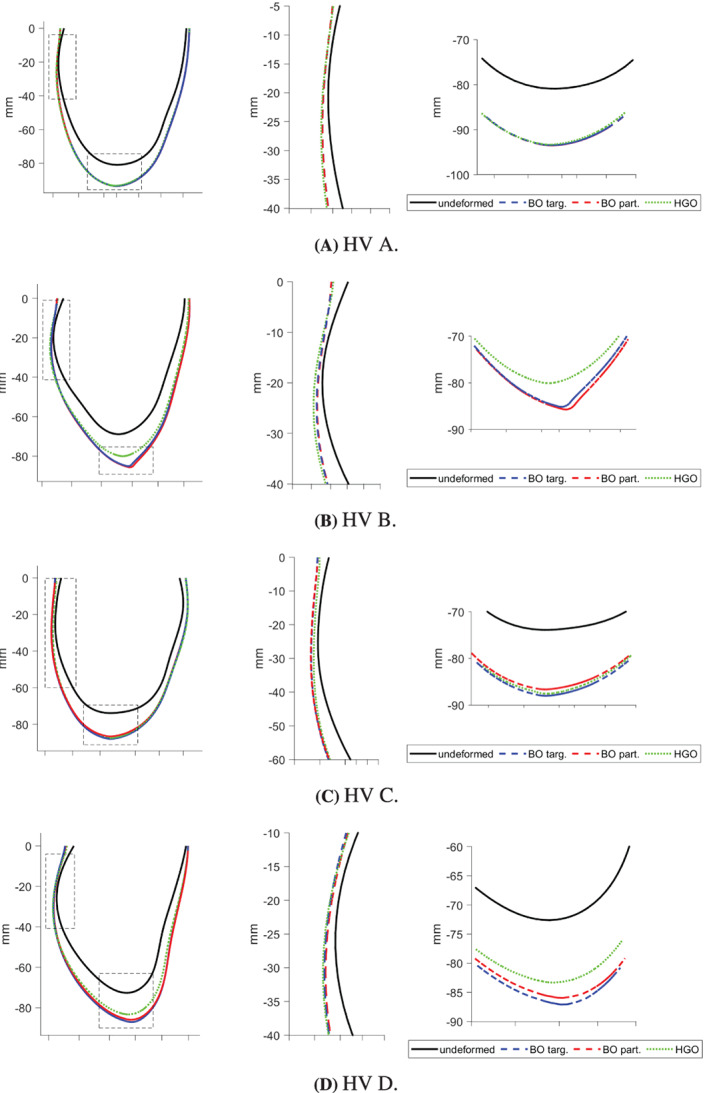
Basic study: deformations of the LV geometry *at 8 mmHg* implied by the final estimates from different algorithms. Left panels show a deformed reference line in the epicardial surface, see the red path in the LV geometry in Figure 3; middle and right panels show details of the inflexion point and the apical region, respectively

As mentioned at the beginning of this section, we focus on analysing stretch‐stress curves and deformations of the LV geometry, presented in Figures 5 and 6, and not parameters estimates explicitly (the latter are nevertheless reported in Appendix A). We observe that the HGO algorithm predicts stiffer myocardium for high stretches along myocyte, while for low stretches the responses from BO and HGO are typically very comparable. The only exception is HV C for which HGO predicts much higher stiffness than BO along the whole range of stretches. On the other hand, for HV C both algorithms predict almost identical responses for stretches along the sheet direction. For the remaining subjects sheet direction stretches are associated with higher stiffness predicted by BO compared with HGO. The deformations of the LV geometry implied by the parameter estimates from different algorithms are generally very similar. The biggest differences between BO and HGO can be observed in the apical region (especailly for HV B), while both BO versions lead to comparable deformations.

We conclude that the original HGO algorithm is easily outperformed by both variants of BO. Moreover, the mechanical response predicted by both BO and HGO for high stretches (i.e. > 1.15) can differ substantially. This could be partially explained by the limitation of using in vivo measurements, which are less informative about stiffening effects under high stretch as the myocardium is mostly operating in the linear regime of the stress‐stretch curve in vivo, in particular for healthy LVs with a maximum stretch below 1.2.^62^ The latter confirms that inferring parameters characterising the nonlinear stiffening effects is challenging when based on in vivo data only. Therefore, introducing extra information about pressures higher than the normal end‐diastolic pressure becomes necessary in order to characterise nonlinear responses at higher pressures. In the clinic, this could be done through stress CMR.^63^ In this study, in our next application we will rely on the Klotz‐law discussed in Section 2.2, which provides an alternative way to capture the nonlinear relationship between the LV cavity volume and the loading pressure within a large range, that is up to as high as 30 mmHg.

### 4.4 Klotz‐curve prior study

In our analysis of the basic study we have demonstrated that our proposed BO framework outperforms the original HGO algorithm^28^ in terms of delivering lower values of the objective function using less invocations of the forward simulator. In this section we want to replace that benchmark with its updated version.^43^ We can now use the full BO algorithm described in Listing 4 as the new HGO algorithm includes the Klotz‐curve prior. This time we run each version of BO (with a target surrogate and a partial error surrogate (13), see Sections 3.1 and 3.2.3), three times (with independent runs based on the same initial design) to verify the robustness and stability of the procedure.

**FIGURE 7:**
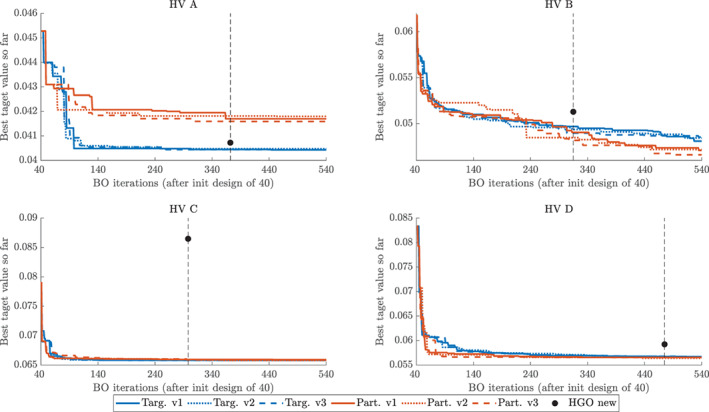
Klotz‐curve study: convergence of the objective function $f_{O2,\text{Klotz}}$ for Bayesian optimisation and the original HGO algorithm for four LV geometries (HV A, HV B, HV C, HV D). Horizontal axis: Bayesian optimisation iterations after 40 iterations for the initial design. Vertical axis: best value of the objective function *f*_*O*2_ recorded so far. Black dot and horizontal dashed line: the final value of the objective function *f*_*O*2_ for the HGO algorithm and the associated number of iterations. Bayesian optimisation with a target surrogate (targ.) and a partial error surrogate (part.), three independent runs (v1, v2, v3) in each version, together with the new version of the HGO algorithm (HGO new). For HGO, the Klotz curve error was computed using the forward simulator (not the emulator)

Figure 7 shows that BO again outperforms the HGO algorithm in terms of achieving lower values of the objective function $f_{O2,\text{Klotz}}$ in fewer iterations. We can see that the updated HGO algorithm performs better than the original one *relatively* to BO, for example for HV C and HV D the new HGO algorithm converges closer to BO than in the basic study. Appendix B provides additional insights into the behaviour of the objective function $f_{O2,\text{Klotz}}$ by decomposing it into the *f*_*O*2_ component (i.e. errors for the 8 mmHg volume and strains) and the Klotz component (i.e. error for the 30 mmHg volume).

**FIGURE 8:**
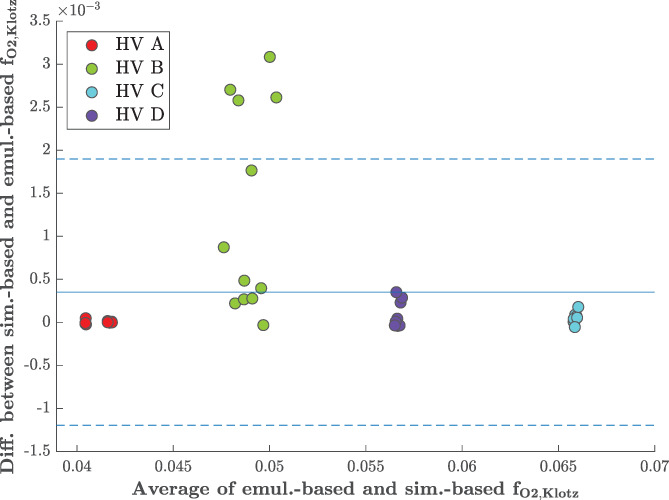
Klotz‐curve: Bland–Altman plot comparing the Klotz objective function with *V*_30_ computed using the emulator and the forward simulator

Table 4 summarises the results related to the convergence of the objective function. Since in the present study the performance of the HGO algorithm is more comparable to the one of BO, we decided to investigate whether the low values of the objective function for BO were not a consequence of a potential bias in the *V*_30_ emulator. The latter is used as a part of our framework aimed at achieving computational savings as discussed in Section 3.3, however the new HGO algorithm still uses the forward simulator to obtain the volume at 30 mmHg. We thus run the forward simulator for each run of BO at points θ corresponding to $y_{\min}$ and $y\left(i_{\min}^{\mathrm{HGO}}\right)$, to obtain the true values of *V*_30_ at these points and hence true values of $f_{O2,\text{Klotz}}$. We report the volumes in the columns indicated with *V*_30_, with the *E*(·) values being the volumes from the emulator and the *S*(·) values being the volumes from the simulator. Similarly, we have two sets of the objective function values, obtained with the simulator (with superscript *S*) and the emulator. These results reveal that our *V*_30_ emulator provides very accurate predictions, with hardly any differences in the high pressure volumes and consequently in the values of the objective function compared to the simulator. Figure 8 illustrates this point using a Bland–Altman plot. The first four sets of points (from the left) show the differences between the simulator‐based and emulator‐based values of $f_{O2,\text{Klotz}}$ against the average over both these values. Note that the scale of the *y*‐axis is 10^−3^. We can see that all the differences are indeed negligible so comparing the HGO algorithm against emulator‐based runs of BO is valid.

**TABLE 4:** Klotz‐curve study: convergence of the objective function for Bayesian optimisation and the updated HGO algorithm for four LV geometries (HV A, HV B, HV C, HV D)

HV	Algorithm/run	$y_{\min}$	$y_{\min}^{S}$	$i_{\min}$	$y\left(i_{\min}^{\mathrm{HGO}}\right)$	$y^{S}\left(i_{\min}^{\mathrm{HGO}}\right)$	$i\left(y_{\min}^{\mathrm{HGO}}\right)$	$E\left(y_{\min}\right)$	$S\left(y_{\min}\right)$	$E\left(i_{\min}^{\mathrm{HGO}}\right)$	$S\left(i_{\min}^{\mathrm{HGO}}\right)$	#Cr.
Target surrogate												
HV A	BO v1	0.040	0.040	443	0.040	0.040	97	223	222	224	223	3
	BO v2	0.040	0.040	248	0.040	0.040	110	225	224	225	224	2
	BO v3	0.040	0.040	258	0.040	0.040	107	224	223	224	223	3
	HGO new	0.041	0.041	371	0.041	0.041	371	–	224	–	224	0
HV B	BO v1	0.048	0.048	526	0.050	0.050	116	189	187	188	188	0
	BO v2	0.048	0.049	510	0.049	0.050	114	189	186	188	186	0
	BO v3	0.049	0.049	534	0.049	0.049	111	189	187	187	186	0
	HGO new	0.051	0.051	314	0.051	0.051	314	–	179	–	179	0
HV C	BO v1	0.066	0.066	140	0.066	0.066	40	229	227	229	227	1
	BO v2	0.066	0.066	327	0.066	0.066	40	230	227	230	227	1
	BO v3	0.066	0.066	192	0.066	0.066	40	230	228	230	228	0
	HGO new	0.086	0.086	297	0.086	0.086	297	–	200	–	200	0
HV D	BO v1	0.057	0.057	534	0.057	0.057	95	152	149	152	149	0
	BO v2	0.057	0.057	464	0.057	0.057	85	153	149	153	149	2
	BO v3	0.057	0.057	500	0.057	0.057	92	153	150	153	150	1
	HGO new	0.059	0.059	474	0.059	0.059	474	–	144	–	144	0
Partial error surrogate												
HV A	BO v1	0.042	0.042	363	0.042	0.042	∅	223	223	223	223	1
	BO v2	0.042	0.042	453	0.042	0.042	∅	224	224	222	222	2
	BO v3	0.042	0.042	379	0.042	0.042	∅	224	225	223	223	1
	HGO new	0.041	0.041	371	0.041	0.041	371	–	224	–	224	0
HV B	BO v1	0.047	0.050	534	0.049	0.052	97	185	179	182	178	1
	BO v2	0.047	0.048	449	0.048	0.052	208	188	184	183	177	1
	BO v3	0.047	0.049	486	0.048	0.050	88	185	179	184	180	0
	HGO new	0.051	0.051	314	0.051	0.051	314	–	179	–	179	0
HV C	BO v1	0.066	0.066	446	0.066	0.066	40	229	227	229	227	5
	BO v2	0.066	0.066	357	0.066	0.066	40	229	227	228	226	1
	BO v3	0.066	0.066	295	0.066	0.066	40	231	228	231	228	0
	HGO new	0.086	0.086	297	0.086	0.086	297	–	200	–	200	0
HV D	BO v1	0.057	0.057	330	0.057	0.057	55	152	150	152	150	0
	BO v2	0.056	0.057	434	0.056	0.057	58	151	148	151	148	2
	BO v3	0.057	0.056	382	0.057	0.056	73	153	150	153	150	3
	HGO new	0.059	0.059	474	0.059	0.059	474	–	144	–	144	0

*Note*: Three independent runs for BO (v1, v2, v3) together with the new version of HGO (HGO new). Missing values for simulator‐based values ($y_{\min}^{S}$, $V_{30}$ with $S\left(y_{\min}\right)$ and $S\left(i_{\min}^{\mathrm{HGO}}\right)$) when the simulator crashed.

**FIGURE 9:**
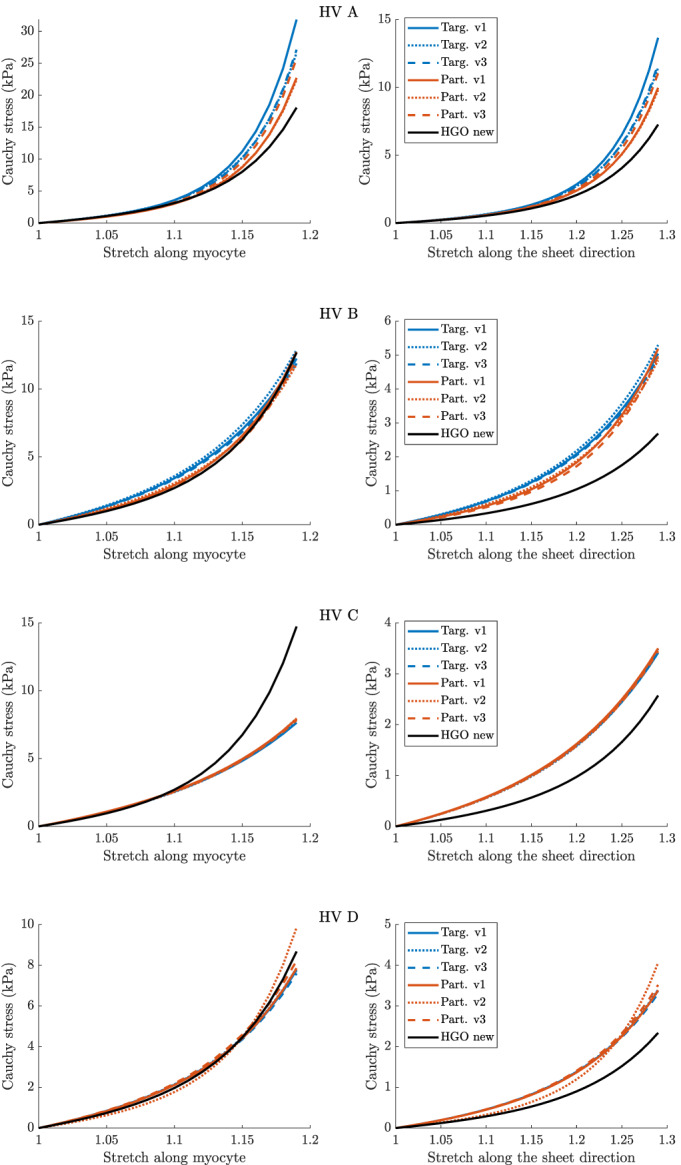
Klotz‐curve study: stretch‐stress curves for four LV geometries (HV A, HV B, HV C, HV D). Left: responses to stretches along the myocyte direction $f_{0}$, right: responses to stretches along the sheet direction $s_{0}$ (see (2)). Bayesian optimisation with a target surrogate (targ.) and a partial error surrogate (part.), three independent runs (v1, v2, v3) in each version

**FIGURE 10:**
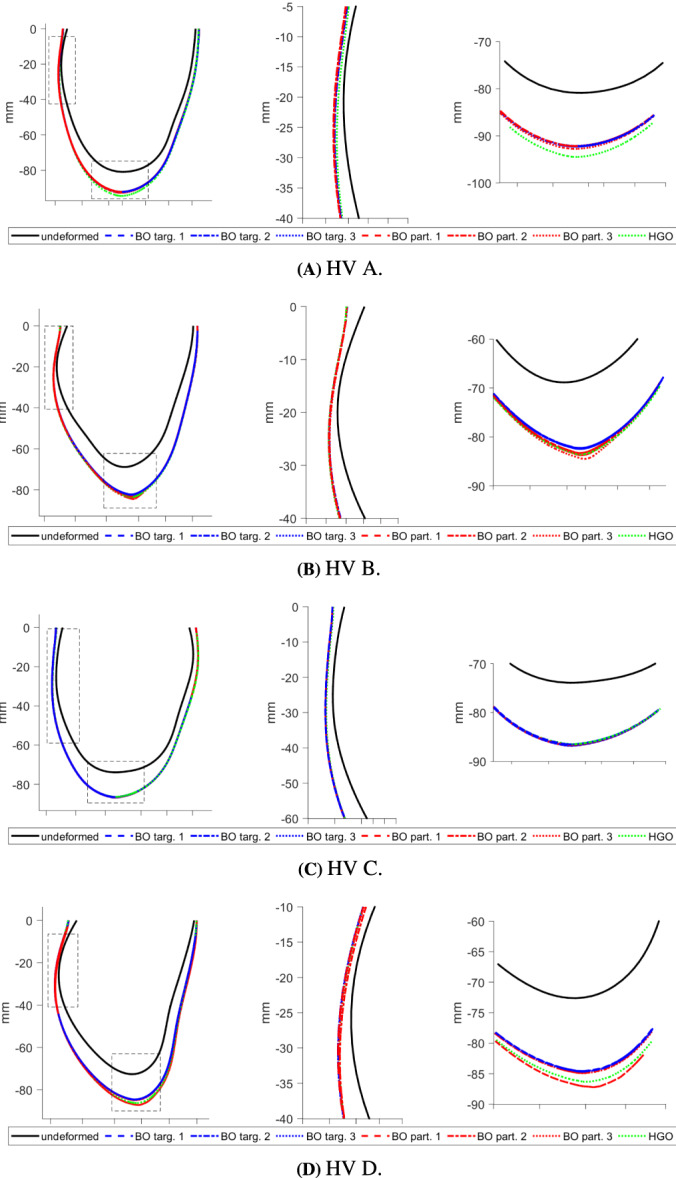
Klotz‐curve study: deformations of the LV geometry *at 8 mmHg* implied by the final estimates from different algorithms. Left panels show a deformed reference line in the epicardial surface, see the red path in the LV geometry in Figure 3; middle and right panels show details of the inflexion point and the apical region, respectively

The stretch‐stress curves in Figure 9 show that including the Klotz‐curve prior leads to the results from BO and HGO being more comparable. For all the curves, except HV C along the sheet direction, the differences in predictions for small stretches (observable in vivo) observed in the basic study now with the Klotz‐curve prior are considerably reduced. Moreover, comparing for example the curves along the myocyte direction for HV B with those from Figure 5 we observe that now the predicted stiffness for high stretches is noticeably reduced. A similar observation can be made for the deformations of the LV geometry, see Figure 10. For instance the difference in the apical region for HV B visible in Figure 6 have now disappeared.

## 5 DISCUSSION

### 5.1 Surrogates performance and heterogeneity

Comparing the performance of target and partial error surrogates does not reveal a clear pattern. In fact, the preferred surrogate type turns out to be case‐specific (depending on the LV geometry and the data), so that one cannot know a priori which surrogate to use. Therefore, for robustness and extra uncertainty quantification, we would suggest trying both approaches, preferably in parallel.

Finally, we note that we would expect partial error surrogates to be superior to target surrogates when there is considerable heterogeneity in the strain data, which would be the case for MI patients. Logically, in a perfectly homogeneous case *K* partial surrogates would just learn the same mapping, which would lead to *K* same surrogates. The more heterogeneity in the strain data, the more need for flexibility in modelling individual errors.

### 5.2 Acquisition function

The particular choice of the acquisition function is pivotal for the performance of BO. The Expected Improvement (EI) criterion applied in this paper has a number of advantages, most importantly it admits a closed form formula under Gaussian process surrogates and is widely used in the machine learning community. However, we note that the analytic tractability of EI becomes less advantageous as we do not use just EI as our AF, but ${\mathrm{EI}}_{\mathrm{con}}$, in which EI is augmented with the probit‐based probability of unknown constraint satisfaction, making the whole AF not tractable. Moreover, EI might perform in a greedy way.^64^

The development of better specifications for acquisition functions is an active research area in the BO literature.^6^ Two classes of alternative acqusition functions seem to be particularly relevant in our context. The first group consists of information‐based policies, such as the Entropy Search^65^, ^66^ (ES) criterion, that aim to infer the location of the unknown minimum by focusing on the posterior distribution of the minimiser. When deciding where to query ES takes into account what we expect to *learn* about the unknown minimum from evaluating at a particular point. This is different to EI, which evaluates where we believe the minimum is, without taking into account the effect of such a query on learning. ES is thus conceptually very appealing, however it is more challenging to implement in practice than EI. The second group of potentially useful AF is related the so‐called portfolios of AFs.^6^, ^67^ As demonstrated by our empirical studies, it is unlikely to develop a globally preferable acquisition strategy, that is preforming best regardless of the given LV geometry and measurements. The portfolio approach considers multiple AFs and uses a meta‐criterion (a high level AF) to select the final query point from those proposed by individual AFs.

### 5.3 Computing time

Depending on the hardware used and the number of parallel tasks performed on a given clusters, computing time per BO iteration may vary considerably. For our studies, the mean computing time was approximately 9.5 minutes (on a typical Linux workstation being Dual 10 core Intel(R) Xeon(R), with a 2.50 GHz CPU and 64 GB RAM), while the median computing time was slightly over 7 minutes (the 10%, 25%, 75% and 90% quantiles were 5.3, 5.8, 10.8, 16.9 minutes, respectively). In our studies we considered 500 BO iterations, which for the median computing time would correspond to 2.5 days to complete optimisation. However, we note that premature convergence might be satisfactory in the clinic, as we observe rapid convergence of the objective function in the initial iterations. If we conservatively assume 200 BO iterations, that would correspond to 1 day for optimisation with the median computing time.

Therefore, despite considerable efficiency gains compared to the HGO algorithm, BO is still likely to be too time‐consuming to provide a viable tool for the clinical practice if optimisation is supposed to be performed independently for each subject, starting from scratch. Multi‐task BO^68^ could address this issue by leveraging prior knowledge acquired from searches over comparable domains, in our case from optimisation performed for previous subjects. Multi‐task BO uses the data coming from different searches by processing them simultaneously and constructing a multi‐task GP^55^ to capture correlation between related tasks. This approach would allow the user to focus on parameter regions that have been found to be promising.

An alternative approach for fast parameter estimation would be to estimate parameters directly from clinical images without first obtaining strain or displacements using cardiac models, however, to our best knowledge, we are not aware of such studies. Cutting‐edge physics‐informed machine learning approaches may be the answer to that question.^69^, ^70^, ^71^ Nevertheless, we note that the goal of our group's research agenda is to make the whole inference pipeline automatic, that is to estimate the myocardial properties directly from CMR images. One of the stepping stones towards that goal was our work on an automatic framework for LV geometry prediction directly from CMR images based on convolutional neural networks.^72^

### 5.4 Biomechanics cardiac modelling

In this study, we have focused on the passive parameter estimation of in vivo hearts by only using conventional cine CMR images. The main reason is that knowing the mechanical properties of myocardial tissue is essential to understand the underlying mechanisms of various heart diseases, such as diastolic heart failure,^73^ and it can further lead to personalised patient treatments, for example de‐stiffening the heart.^74^ In this study, we have adapted the framework of Bayesian optimisation to infer unknown parameters in the HO model, which outperformed the multi‐step algorithm of Gao et al.^28^ Future studies shall take into account the stiffness heterogeneity and material properties of different constituents that will provide a closer link to cardiac pathology compared to the global stiffness estimation. We also expect that the developed procedure in this study can be readily applied to other inverse problems by simply updating the loss function according to the available measurements, that is nonlinear‐mechanics in general, or any parametric mathematical model.

A standalone single ventricle model during diastolic filling is chosen here by only including the essential features, such as subject‐specific geometry, layered myofibre architecture, etc. Such standalone LV model is still being widely used in cardiac modelling communities.^30^, ^34^, ^75^ It shall be mentioned that the LV model can be further improved by including the right ventricle, the valves, the pericardium with more realistic boundary conditions, and further coupled with the blood flow and ectrophysiology. Such a complex model will inevitably introduce extra unknown parameters, which make the inference problem more challenging. For example, Strocchi et al.^76^ used spatially varying robin boundary conditions to represent the pericardium in a four‐chamber heart model; their results have shown that even though the pericardium can significantly affect ventricular motion, it has little effect on the local strains and wall thickness. The basal plane of the LV model is fixed because of lack of measured cardiac motion on the basal plane in our 2D cine CMR imaging dataset. This could be overcome by 3D tagged CMR imaging,^29^ while it requires complex processing and extra scan time that is not routinely available.

Studies have demonstrated the high descriptive and predictive capabilities of the HO model in modelling cardiac dynamics.^33^, ^34^, ^39^ Due to the poor identifiability of some parameters in the original HO model,^32^ a few reduced HO models have been proposed in the context of parameter inference, such as the reduced formula with transverse isotropy used in Asner et al.^29^ For example, by using a reduced HO model with only the first two terms, Hadjicharalambous et al.^30^ inversely estimated a and $a_{f}$ by fixing *b* and $b_{f}$, and further proposed to learn the ratio $a/a_{f}$. Instead, other studies introduced scaling parameters to simplify the parameter inference problem for the HO model. For example, Peirlinck et al.^34^ introduced two scaling factors for a's and *b* ^*'*^s, respectively. This is the first step in the HGO algorithms. Considering that the HO‐type material models are still being used widely in the cardiac modelling community, the Bayesian optimisation framework developed in this study can be readily extended to other HO‐type strain energy functions, and further to other types of constitutive laws in soft tissue mechanics, such as the Fung‐type strain energy function.^58^

A further future research direction is to estimate spatially varied material stiffness for a given patient, especially for a patient with localised disease, such as myocardial infarction. It is believed that even for a healthy heart, myocardial stiffness will vary spatially. However, there is a lack of controlled experimental data to inform how heterogeneous stiffness is distributed in the myocardium. Should it be based on the widely used AHA‐17 divisions or other segmental divisions (free wall, septum, etc.)? Potentially, CMR T1 mapping may provide key information about interstitial fibrosis,^77^ which could further inform myocardial stiffness. For patients with myocardial infarction, experiments have demonstrated that the infarcted region, in general, has much higher stiffness than healthy myocardium. To model that, regional varied stiffness is usually used when modelling myocardial infarction.^3^ In a recent study, Balaban et al.^78^ have demonstrated that heterogeneous elastic material properties in infarcted hearts could be estimated by using an adjoint‐based data assimilation approach with a reduced HO model. Further validation studies with controlled experiments are needed. For patients with diastolic heart failure,^23^, ^73^ the heart remodels its function and structure more globally compared to myocardial infarction, including the remodelling of myocardial stiffness, thus the global myocardial stiffness, as estimated in this study, is still relevant to those patients.

To sum up, this study should be considered as a step forward for passive parameter identification in cardiac mechanics using the HO model. Still many challenges need to be overcome before addressing the passive parameter identification in full for modelling cardiac mechanics, such as detailed cardiac motion measurements, unloaded residual‐stressed geometry, micro‐structure informed spatial heterogeneity, interactions with active contraction and electrophysiology, in vivo experiments, etc. Interested readers are referred to recent reviews in computational cardiac mechanics.^1^, ^79^

## 6 CONCLUSIONS

We have proposed an efficient, Bayesian optimisation–based framework for parameter inference in a cardiac mechanic model of the left ventricle in diastole. We have demonstrated that BO converges to lower values of the two objective functions considered and requires less invocations of the associated forward simulator than established state‐of‐the‐art iterative optimisation algorithms. We have developed a new approach to minimising a target function given as a sum of error terms based on approximating each of these terms individually via partial error surrogates. The findings of our empirical studies suggest that this approach may outperform the standard one based on target surrogates. However, which of the two variants is preferred in a specific case could be subject‐dependent (depending on e.g. the LV geometry).

## CONFLICT OF INTEREST

The authors declare no potential conflict of interests.

## Figures and Tables

**FIGURE B1:**
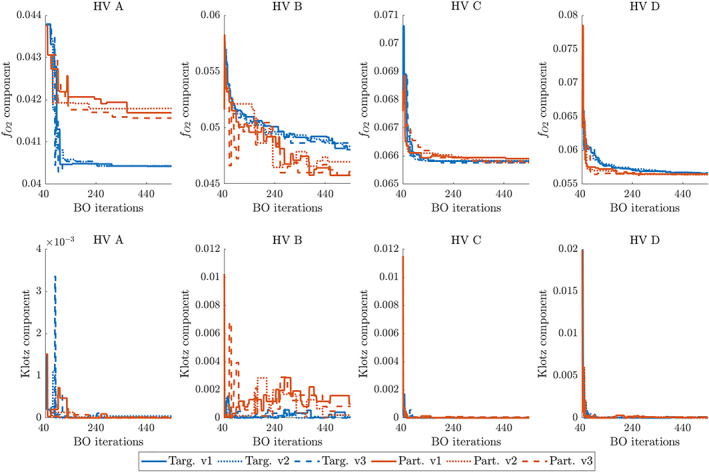
Klotz study: decomposition of the incumbent trajectories from Figure 7 based on $f_{O2,\text{Klotz}}$ from (11) into $f_{O2}$ from (6) (top) and the Klotz component (bottom). Bayesian optimisation with a target surrogate (targ.) and a partial error surrogate (part.), three independent runs (v1, v2, v3) in each version

**FIGURE C2:**
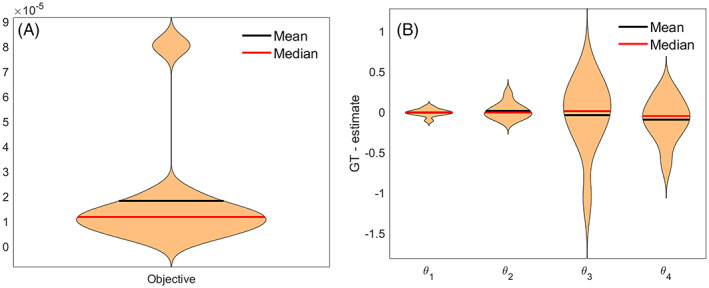
Synthetic data study: estimation errors. Left: objective function; right: parameters

**FIGURE C3:**
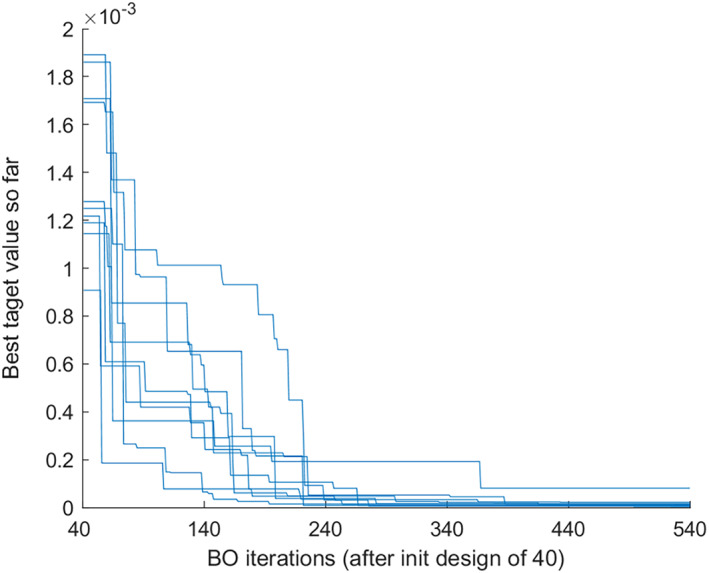
Synthetic data study: incumbent convergence for ten synthetic data sets

**FIGURE D4:**
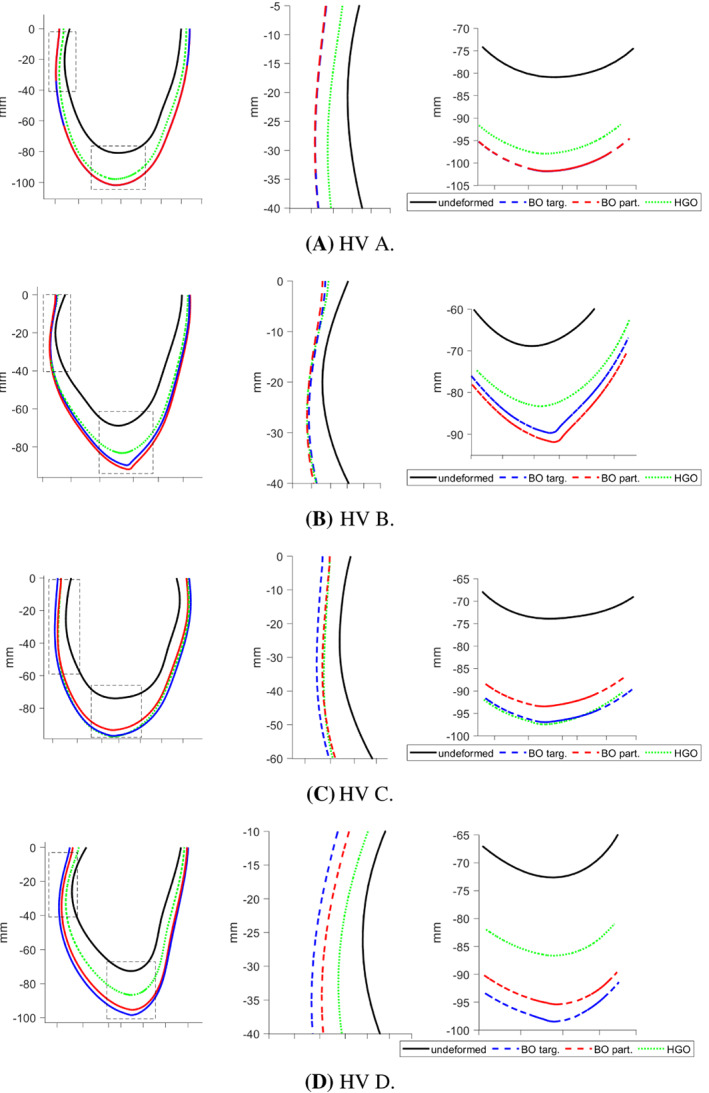
Basic study: deformations of the LV geometry *at 30 mmHg* implied by the final estimates from different algorithms. Left panels show a deformed reference line in the epicardial surface, see the red path in the LV geometry in Figure 3; middle and right panels show details of the inflexion point and the apical region, respectively

**FIGURE D5:**
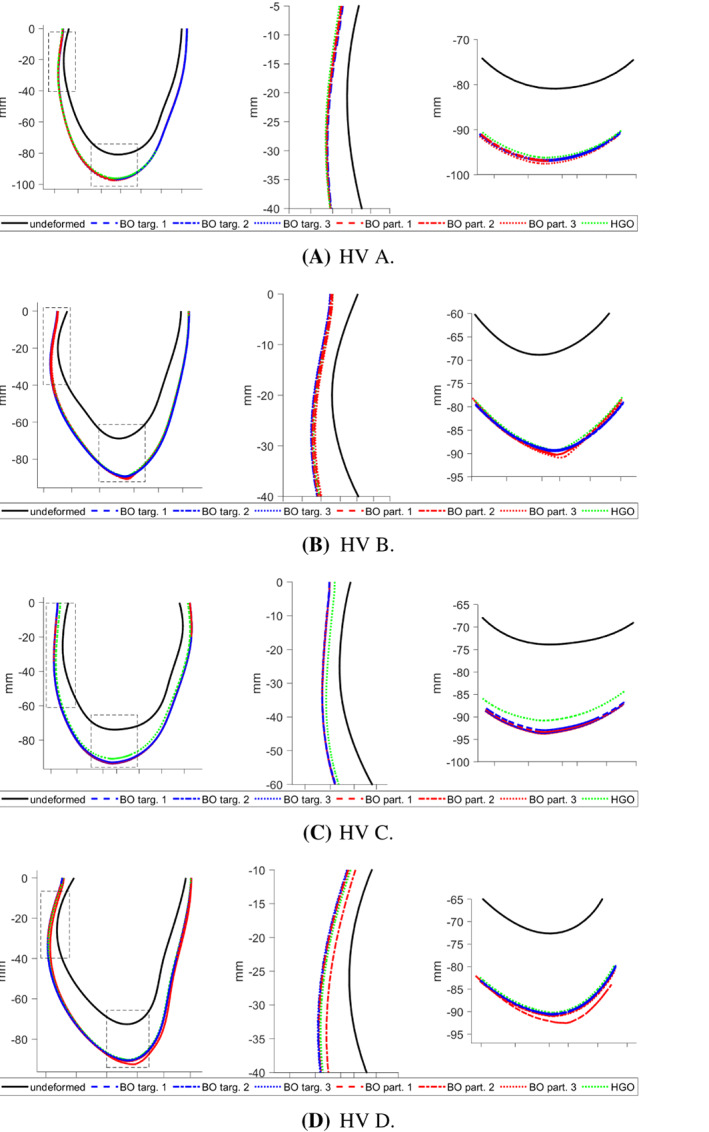
Klotz‐curve study: deformations of the LV geometry *at 30 mmHg* implied by the final estimates from different algorithms. Left panels show a deformed reference line in the epicardial surface, see the red path in the LV geometry in Figure 3; middle and right panels show details of the inflexion point and the apical region, respectively

**FIGURE E6:**
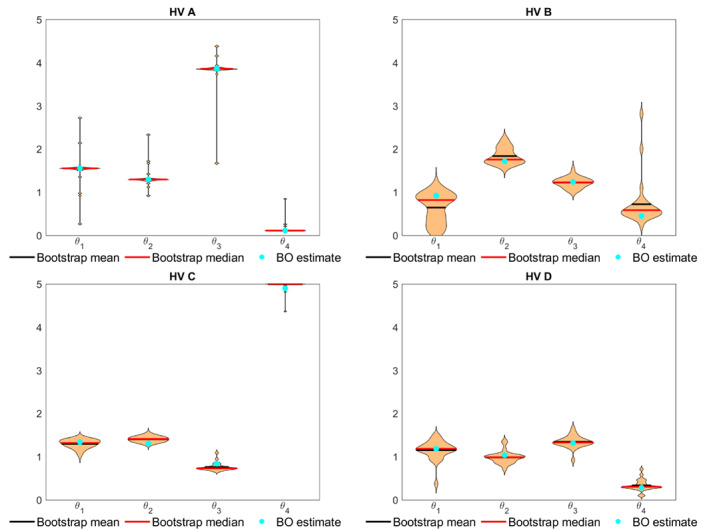
Klotz‐curve study: violin plots for the bootstrapped parameter estimates from run v1 with target surrogates

**FIGURE F7:**
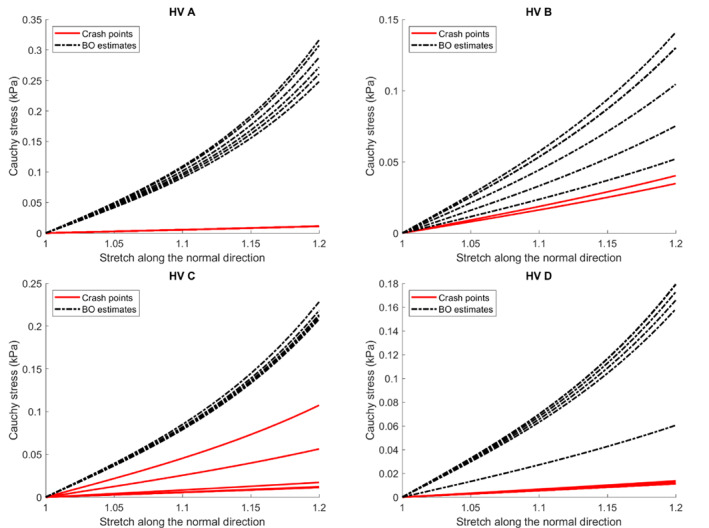
Uni‐axial stretch‐stress responses along the sheet‐normal direction for crash points (solid red) and the BO estimates (dashed black)

**FIGURE G8:**
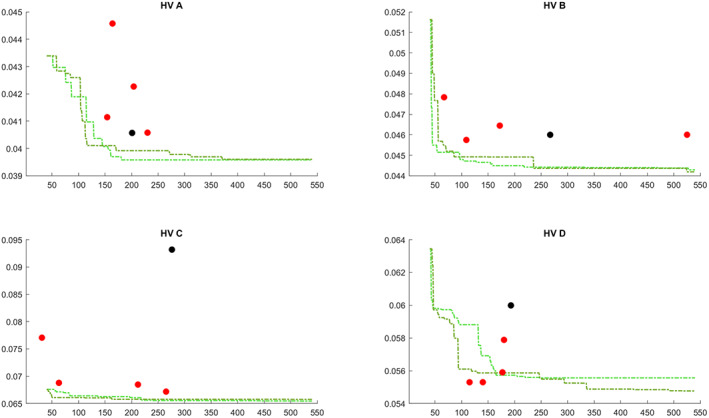
Basic study: comparison of the BO convergence (dashed lines, light green: target surrogates; dark green: partial error surrogates) with the final optima from the SQP (four red dots, one for each initial point) and HGO (black dot) algorithms

## APPENDIX A RESULTS ON PARAMETERS

### A.1

#### A.1 Basic study

Table A1.

**TABLE A1:** Basic setting: final optimised values of the eight parameters of the HO law for Bayesian optimisation and the original HGO algorithm (HGO old) for four different LV geometries (HV A, HV B, HV C, HV D), Bayesian optimisation with a target surrogate (targ.) and a partial error surrogate (part.)

HV	Algorithm	a	b	$a_{f}$	$b_{f}$	$a_{s}$	$b_{s}$	$a_{\mathrm{fs}}$	$b_{\mathrm{fs}}$
HV A	BO targ.	0.257	3.715	8.636	0.409	1.784	0.166	0.045	0.371
	BO part.	0.258	3.728	8.581	0.360	1.773	0.146	0.074	0.619
	HGO old	0.100	5.405	6.172	7.114	1.117	2.582	0.123	5.650
HV B	BO targ.	0.018	0.260	3.174	7.859	0.656	3.196	1.444	12.016
	BO part.	0.018	0.260	4.344	4.780	0.898	1.944	1.458	12.131
	HGO old	0.100	5.766	3.090	11.252	0.372	2.582	0.041	5.650
HV C	BO targ.	0.207	2.991	5.567	0.294	1.150	0.119	1.546	12.864
	BO part.	0.245	3.532	4.481	2.158	0.926	0.877	1.550	12.900
	HGO old	0.100	1.960	8.447	1.811	1.117	0.516	0.123	1.130
HV D	BO targ.	0.162	2.342	4.834	0.293	0.999	0.119	0.676	5.623
	BO part.	0.174	2.519	4.982	0.878	1.029	0.357	0.169	1.406
	HGO old	0.100	5.405	2.626	8.791	0.372	2.582	0.041	5.650

#### A.2 Klotz‐curve study

Table A2.

**TABLE A2:** Klotz‐curve study: final optimised values of the parameters of the HO law for Bayesian optimisation and the updated HGO algorithm for four LV different geometries (HV A, HV B, HV C, HV D)

HV	Algorithm/run	a	b	$a_{f}$	$b_{f}$	$a_{s}$	$b_{s}$	$a_{\mathrm{fs}}$	$b_{\mathrm{fs}}$
Target surrogate									
HV A	BO v1	0.279	4.033	4.326	10.536	0.894	4.284	0.034	0.287
	BO v2	0.288	4.154	4.530	9.225	0.936	3.751	0.119	0.994
	BO v3	0.299	4.319	4.307	9.624	0.890	3.913	0.031	0.258
	HGO	0.205	6.404	4.411	7.123	0.819	2.912	0.368	6.767
HV B	BO v1	0.166	2.395	5.727	3.382	1.183	1.375	0.139	1.156
	BO v2	0.166	2.398	5.977	3.432	1.235	1.395	0.039	0.324
	BO v3	0.177	2.554	5.605	3.329	1.158	1.353	0.120	0.999
	HGO	0.100	2.113	4.126	5.506	0.555	1.655	0.250	3.847
HV C	BO v1	0.254	3.673	4.463	1.995	0.922	0.811	1.550	12.899
	BO v2	0.241	3.476	4.357	2.287	0.900	0.930	1.519	12.640
	BO v3	0.242	3.496	4.515	2.156	0.933	0.877	1.548	12.886
	HGO	0.126	4.098	3.897	6.689	0.481	1.750	0.216	4.067
HV D	BO v1	0.213	3.075	3.458	3.591	0.714	1.460	0.087	0.727
	BO v2	0.207	2.989	3.479	3.641	0.719	1.480	0.062	0.515
	BO v3	0.213	3.070	3.473	3.446	0.718	1.401	0.061	0.511
	HGO	0.108	3.618	3.043	5.041	0.458	1.684	0.206	3.915
Partial error surrogate									
HV A	BO v1	0.319	4.605	3.819	9.279	0.789	3.773	0.619	5.150
	BO v2	0.313	4.516	3.977	8.964	0.822	3.645	0.660	5.489
	BO v3	0.270	3.897	4.046	9.684	0.836	3.937	0.586	4.875
	HGO	0.205	6.404	4.411	7.123	0.819	2.912	0.368	6.767
HV B	BO v1	0.105	1.521	4.564	4.942	0.943	2.009	0.515	4.287
	BO v2	0.139	2.007	4.958	4.018	1.024	1.634	0.452	3.764
	BO v3	0.076	1.101	4.274	5.112	0.883	2.078	0.628	5.227
	HGO	0.100	2.113	4.126	5.506	0.555	1.655	0.250	3.847
HV C	BO v1	0.246	3.555	4.536	2.121	0.937	0.862	1.433	11.930
	BO v2	0.238	3.432	4.398	2.335	0.909	0.950	1.550	12.900
	BO v3	0.239	3.446	4.407	2.225	0.910	0.905	1.509	12.562
	HGO	0.126	4.098	3.897	6.689	0.481	1.750	0.216	4.067
HV D	BO v1	0.201	2.897	3.421	3.734	0.707	1.518	0.175	1.457
	BO v2	0.087	1.258	2.534	6.858	0.523	2.789	0.304	2.531
	BO v3	0.194	2.797	3.534	3.821	0.730	1.554	0.130	1.080
	HGO	0.108	3.618	3.043	5.041	0.458	1.684	0.206	3.915

*Note*: For each BO variant (target surrogate, partial error surrogate three independent runs are reported (v1, v2, v3).

## APPENDIX B DECOMPOSITION OF $f_{O2,\text{Klotz}}$ INTO $f_{O2}$ AND THE KLOTZ COMPONENT

Figure B1 decomposes the incumbent convergence plot from Figure 7 into the $f_{O2}$ component, that is errors for the 8 mmHg volume and strains, (top) and the Klotz component, that is error for the 30 mmHg volume (bottom). When compared with Figure 4 from the Basic study in Section 4.3  we can see that the $f_{O2}$ component is larger in the Klotz study (Section 4.4). Besides, the magnitude of the Klotz component quickly becomes negligible compared to the $f_{O2}$ component

## APPENDIX C SYNTHETIC DATA STUDY

In this appendix, we report the results of a synthetic data study aimed at verifying the theoretical accuracy of the proposed method. We consider the geometry of HV A and generate ten synthetic data sets by perturbing the estimate for HV A corresponding to the lowest value of the objective function with Gaussian noise. This way we obtain synthetic data sets with known underlying ground‐truth (GT) parameter values, which we then estimate with BO.

Figure C2 presents the results from this experiment in terms of errors in the output space (by means of the objective function) and in the parameter space (by means of the difference between the GT value and its estimate). Figure C3 illustrates the convergence of the incumbent values. We can see that the convergence of the objective function is rapid and to very low values (compared with the real data studies in Sections 4.3  and 4.4), of order 10^−5^. Moreover, we notice that on average the difference between the GT parameter values and their estimates is zero. As expected, $\theta_{1}$ and $\theta_{2}$ are estimated with high precision, while $\theta_{3}$ and $\theta_{4}$ are harder to identify, hence there is more variance in the corresponding estimation errors.

## APPENDIX D LV GEOMETRY DEFORMATIONS

Figures D4 and D5 provide high‐pressure (at 30 mmHg) counterparts of low pressure (8 mmHg) Figures 6 and 10, respectively. Applying much higher pressure results in differences between deformations implied by estimates from different algorithms being exaggerated. Still, the deformations implied by BO and HGO estimates are generally consistent, with most noticeable differences appearing in the apical region.

## APPENDIX E UNCERTAINTY QUANTIFICATION

Recently, there has been an increasing awareness in the cardiac modelling community of the importance of uncertainty quantification and sensitivity analysis for model parameter estimates.^59^, ^60^, ^61^ Below we describe three principled directions one can take to assess the uncertainty of parameter estimates: an asymptotic approach, a computational frequentist approach, and a computational Bayesian approach. We also discuss the advantage of Gaussian processes (see Section 3.1) used in this work over polynomial chaos methods in the context of BO.

### E.1 Asymptotic approach

The asymptotic approach is based on the Fisher information inequality and the Cramer‐Rao bound, whereby the variance of an estimated parameter is lower‐bounded by the inverse of the negative second derivative of the log likelihood with respect to this parameter. For additive independent Gaussian noise, the log likelihood is just a rescaled version of our sum‐of‐squares objective function; so it can easily be obtained. To generalise this to more than one parameter, we need to get the diagonal elements of the inverse of the negative Hessian, that is the matrix of negative second derivatives of the log likelihood. To compute the second derivatives, we can exploit the fact that our objective function is approximated by a Gaussian process, and that a Gaussian process is closed under differentiation, that is if the kernel function of the Gaussian process is twice differentiable, we will get these derivatives in closed form. The advantage of this approach is that it is analytically tractable and fast. The disadvantage is that it is based on asymptotic assumptions. These may not hold for sparse data, with the consequence that the lower bound may not be tight. For that reason, we prefer computationally more expensive non‐asymptotic methods.

### E.2 Computational frequentist approach

This approach is based on a residual bootstrapping procedure^80^ to estimate the parameter estimation uncertainty. Take the predictions (standardised circumferential strains and end‐diastole LV volume) obtained from the final parameter vector estimate $\theta^{*},\;\xi \left(\theta^{*}\right)$, and compute the residuals by comparison with the data. Let us call the set of residuals *E*. We then generate an ensemble of hypothetical data, $D_{1},\ldots ,D_{K}$, by drawing residuals with replacement from *E* and adding these values to $\xi \left(\theta^{*}\right)$. For each of these bootstrap replica, $D_{1},\ldots ,D_{K}$, we carry out a local iterative optimisation (e.g. using conjugate gradients), starting from $\theta^{*}$. This procedure returns a set of bootstrap estimates, $\left\{\theta_{1},\theta_{1},\ldots ,\theta_{K}\right\}$, which can be used for uncertainty quantification. We use the median absolute deviation (MAD) as a simple summary statistic; this is a robust measure of variability that is less susceptible to outliers than the standard deviation. We multiply the MAD with a constant scale factor of k = 1.4826, which makes it a consistent estimator for the estimation of the standard deviation of a normally distributed random variable.

Figure E6 presents examples of bootstrap results by means of violin plots for BO estimates (with target surrogates, run v1) from the Klotz‐curve study. Each figure is based on *K* = 25 bootstrap samples. Recall that the parameters come from a compact domain [0.1,5]^4^ hence any apparent truncation of a violin plot (e.g. $\theta_{4}$ for HV C) is caused by the underlying parameter being estimated close to its boundary. We can see that the uncertainty in parameter estimates for HV A is very small as visualised by hardly visible “violins”; we can observe a few outliers, however. In contrast, the uncertainty for HV B is much larger, as depicted by larger “violin” areas. Table E3 reports the associated MADs

**TABLE E3:** Klotz‐curve study: median absolute deviations (MADs) for the bootstrapped parameter estimates from run v1 with target surrogates

Case	*θ*_1_	*θ*_2_	*θ*_3_	*θ*_4_
HV A	0.204	0.140	0.176	0.070
HV B	0.302	0.159	0.087	0.313
HV C	0.081	0.052	0.062	0.071
HV D	0.145	0.100	0.097	0.094

### E.3 Computational Bayesian approach

We can use the emulated objective function to approximate the log likelihood, define appropriate prior distributions for the parameters and apply standard Markov chain Monte Carlo techniques to approximately sample from the posterior distribution. The practical disadvantage is that this is an intrinsically sequential approach, whereas the optimisation runs for the bootstrap approach can be parallelised.

### E.4 Gaussian processes versus polynomial chaos

Polynomial chaos methods have been widely used as an alternative to Gaussian processes for emulation and surrogate modelling.^59^, ^60^, ^61^ However, for Bayesian optimisation, the emulation of the objective function is not sufficient. We also need a reliable estimate of the variance of the approximated objective function, which is needed for the acquisition function (see Section 3.2). Figure 4 in Shahriari et al.^6^ shows several examples of methods that emulate the objective function well, but provide poor estimates of its variance. This will lead to a poor trade‐off of exploration versus exploitation and is unsuitable for Bayesian optimisation. For that reason, we have used Gaussian processes rather than polynomial chaos expansion in our work.

## APPENDIX F SIMULATOR CRASHES

In our applications, crashes are caused by implausible parameter sets, by which we mean that the “crash” parameter sets result in an excessive distortion of the underlying LV geometry, for example, when all parameters are close to the lower bounds, suggesting a very soft myocardium. An increased number of crashes for BO compared to HGO is a consequence that BO is a global optimisation technique, in which exploitation is balanced with *exploration*, while HGO performs mostly local, gradient‐based steps and hence focuses on exploitation. From this point of view, despite their name, crashes are not necessarily something negative, provided an algorithm has a feature that allows for accounting for them (like the classifier in our proposed BO framework, see Section 3.2.2), as they allow us to learn about the feasible parameter set. We emphasise again that the advantage of our global optimisation scheme is a wider exploration of the configuration space and that our model thus becomes aware of these critical regimes. A local greedy optimisation scheme, on the other hand, is by construction “blinkered”, that is so focused on exploitation that it never explores these critical regimes.

Figure F7 presents uni‐axial stretch‐stress responses along the sheet‐normal direction between the crash points (red) and the BO estimates (black). We can see that the material stiffness along the sheet‐normal direction is dramatically lower in crashed simulations than in successful simulations corresponding to BO estimates, in other words, very soft along the sheet‐normal directions for those crashed simulations. This would explain why we observed crashes for certain parameter sets.

## APPENDIX G COMPARISON WITH A STATE‐OF‐THE‐ART NUMERICAL OPTIMISER

We appreciate that the HGO algorithm runs with respect to different parameters than the proposed BO framework and that the number of parameters used by both algorithms is different (in particular, in HGO the number of parameters with respect to which the optimisation is carried out may vary between the algorithm steps). This may raise concerns about the fairness of the BO–HGO comparison. Therefore, in this appendix, we present a comparison of BO against an established iterative optimisation scheme in the same 4‐dimensional parameter space. Specifically, we consider the SQP algorithm (sequential quadratic programming), which is a state‐of‐the art gradient‐based numerical optimiser, implemented in the fmincon function from MATLAB's Optimisation Toolbox. Since the performance of numerical optimisation depends on the initial point, we have run SQP from 4 different initial points: $\theta_{0}^{\left(1\right)}={\left[1,1,1,1\right]}^{T}$ and three points closest to $\theta_{0}^{\left(1\right)}$ in the Latin hypercube initial design used to initialise BO. Figure G8 reveals that BO converges to lower values of the objective function for all the HVs. Moreover, BO typically requires fewer forward simulations achieve the same level of accuracy as the numerical optimizer.
